# Role of Vibronic Coupling for the Dynamics of Intersystem Crossing in Eu^3+^ Complexes: an Avenue for Brighter Compounds

**DOI:** 10.1021/acs.jctc.4c01461

**Published:** 2025-03-07

**Authors:** Leonardo F. Saraiva, Albano N. Carneiro Neto, Airton G. Bispo-Jr., Mateus M. Quintano, Elfi Kraka, Luís D. Carlos, Sergio A. M. Lima, Ana M. Pires, Renaldo T. Moura Jr.

**Affiliations:** † Department of Chemistry (Computational and Theoretical Chemistry Group), Southern Methodist University (SMU), Dallas, Texas 75725, United States; ‡ Department of Chemistry and Biochemistry, School of Science and Technology, São Paulo State University (UNESP), São Paulo, 19060-900, Brazil; § Academic Unit of Cabo de Santo Agostinho, Federal Rural University of Pernambuco (UFRPE), Cabo de Santo Agostinho, 54518-430, Brazil; ⊥ Aveiro Institute of Materials, Physics Department, University of Aveiro, Aveiro, 3810-193, Portugal; ∥ Institute of Chemistry, University of São Paulo (USP), São Paulo, 05508-900, Brazil

## Abstract

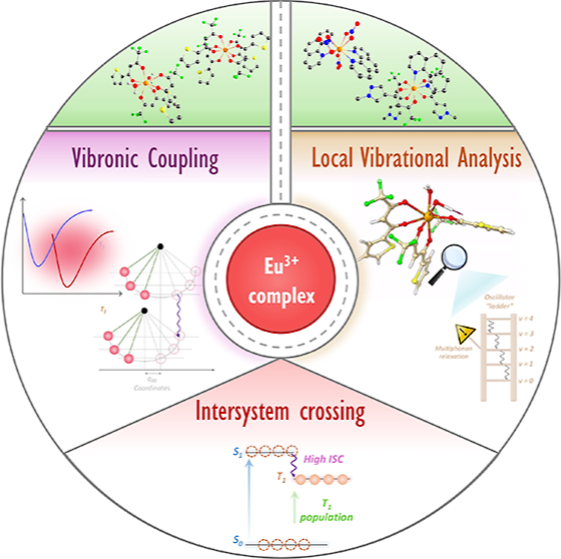

Understanding the dynamics of photophysical processes in Ln^3+^ complexes remains challenging due to the intricate nature involving the metallic center, where sensitization (antenna effect) plays a pivotal role. Current studies have often overlooked the vibronic coupling within the antenna effect, leading to incomplete insights into excited-state dynamics. To address these shortcomings, we introduce a novel theoretical and computational approach that leverages the impact of the vibrational modes of the S_1_ and T_1_ states in this effect through the correlation function formalism, offering a comprehensive view of intersystem crossing (ISC). Our approach achieves a desirable alignment between empirical and theoretical rates, outperforming previously employed semiclassical methods. A groundbreaking finding is that vibronic coupling with vibrations in the 700–1600 cm^–1^ energy range is crucial for higher ISC, and local vibrational mode analysis identified that this process is driven by delocalized vibrations across the molecule. These results shed light on the key molecular fragments responsible for vibronic coupling, opening an avenue for harnessing faster ISC by tailoring the ligand scaffold. Overall, it also demonstrates how ISC dynamics can serve as a bridge between theory and experiment, furnishing detailed mechanistic insights and a roadmap for the development of brighter compounds.

## Introduction

Inspired by the daily use of photonic materials, chemists have made significant strides in the development of compounds with noteworthy photophysical features.^1−3^ These compounds find practical applications in diverse fields such as biomedicine, anticounterfeiting measures, and light-emitting diodes (LEDs).^4−6^ Compounds relying on the luminescence of trivalent lanthanide ions (Ln^3+^)^7,8^ are frequently regarded as suitable building blocks for the design of luminescent materials. This preference stems from their distinctive spectroscopic characteristics, which include a plethora of sharp and well-defined emission bands that span from the ultraviolet (UV) to near-infrared (NIR) spectral range. These bands are directly associated with intraconfigurational 4*f* ↔ 4*f* electronic transitions,^9,10^ yet 4*f* ↔ 5*d* transitions are also possible in some cases.^11^ However, the direct sensitization of 4*f* ↔ 4*f* Ln^3+^ ions encounter challenges associated with low molar absorptivity, which is primarily attributed to the parity-forbidden nature of such transitions when considering the free ion.^12^ To address this challenge, a common strategy involves coordinating organic chromophores with the Ln^3+^ center. In this approach, the organic ligand acts as an “antenna”, absorbing energy from an excitation source and subsequently transferring it to the Ln^3+^ ion.^1^

The sensitization mechanism underlying this process, known as the antenna effect, has been extensively researched and is often elucidated through the following representative steps: (*i*) excitation of organic ligands to the first singlet excited state (S_0_ → S_1_); (*ii*) intersystem crossing (ISC) from the singlet to the low-lying triplet state (S_1_ → T_1_); and (*iii*) intramolecular energy transfer (IET) from the T_1_ (and/or S_1_) to ^2S+1^L_J_ levels of the Ln^3+^ ion.^13^ Based on this mechanism, opportunities for fine-tuning the photoluminescence of Ln^3+^ compounds are created by regulating the energy and population of the S_1_ and T_1_ states of the ligands, particularly those favoring the intramolecular energy transfer process.

Recent studies have endeavored to quantitatively describe each step of the sensitization mechanism^12,14−16^ using well-established modeling frameworks targeting the absorption of organic ligands and intramolecular energy transfer. These efforts often rely on the model developed by Malta,^17,18^ which provides theoretical values for emission quantum yields (QY) that align with empirical data. However, accurately describing the rates involved in the ISC step remains an ongoing challenge.^19^ This difficulty stems from the spin–orbit coupling (SOC) induced by the 4*f* electrons of Ln^3+^ ions,^20,21^ which complicates the estimation of rates through semiclassical models such as the Marcus-Levich eq (eq S1).^22,23^ To overcome these limitations, researchers often resort to approximating the ISC rates to mean values^12,24^ or oversimplifying different methods by solely considering the vertical energy difference between the S_1_ and T_1_ states.^12^ However, this approach frequently results in underestimated ISC rates. The primary reason for this underestimation lies in neglecting vibronic coupling,^25^ where normal modes induce fluctuations in the adiabatic S_1_–T_1_ energy splitting.^26^ Therefore, incorporating vibronic coupling effects allows for a more accurate estimation of ISC, as previously demonstrated in the study of organic molecules and *d*-metal complexes such as xanthone,^27^ [Cu(dppb)(pz_2_bph_2_)],^28^ and [Ir(ppy)_3_].^29^

Considering these findings, it is conceivable to assert that reducing the adiabatic energy splitting between the singlet and triplet states through the mediation of vibrational modes affects the ISC rates in Ln^3+^ complexes.^30^ It is worth noting that high-energy normal modes also serve as a pathway for nonradiative deactivation through vibronic coupling and multiphonon relaxation,^31^ leading to the quenching of Ln^3+^ emission.^32^ However, to the best of our knowledge, no study has explored the role of vibrational coupling on the ISC rates of Ln^3+^ complexes in detail. This raises questions about the overall impact of vibronic coupling on the photophysical features of Ln^3+^ complexes, considering its effect on both ISC and nonradiative deactivation mechanisms. Therefore, offering accurate models and conducting an in-depth analysis of the role of vibronic coupling would undoubtedly advance our understanding of the excited-state processes of Ln^3+^ complexes. In addition, such an analysis could provide important data on how to tune the luminescence of these compounds by tailoring the ligand scaffold. These advances enable the enhancement of their photophysical features, facilitating the design of novel luminescent materials that can be used in state-of-the-art photonic devices.

**Scheme 1:**
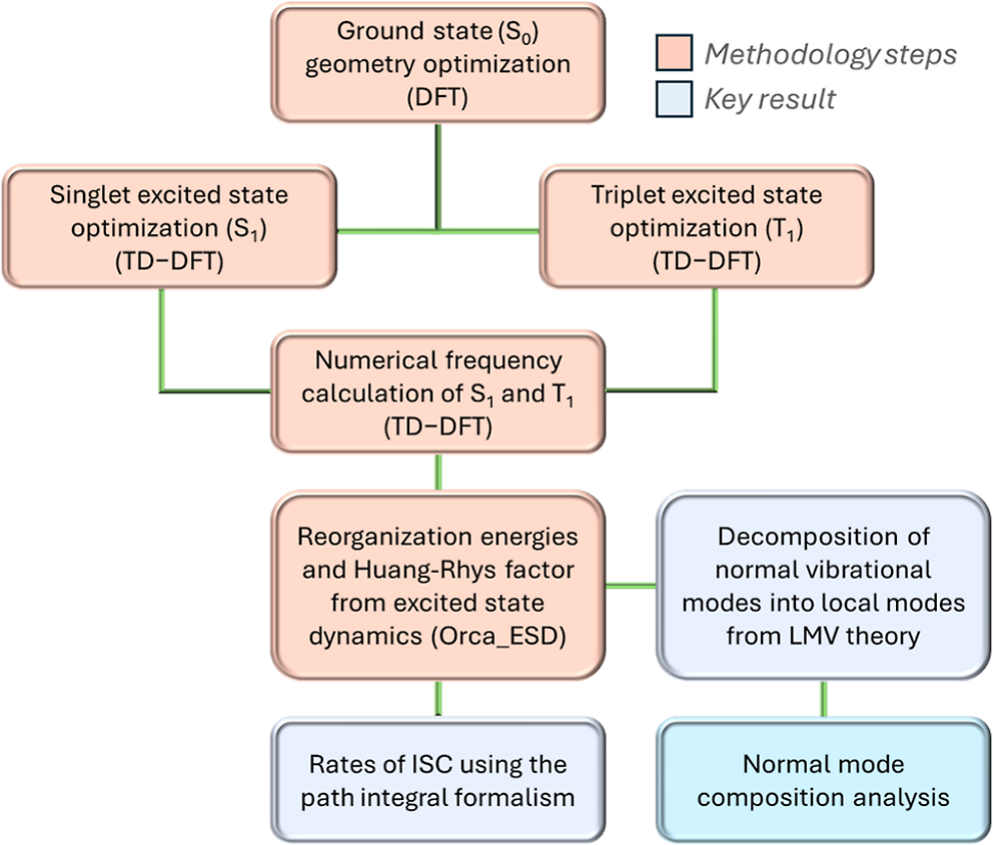
Conceptual Flowchart of the Methodology of the Proposed Approach All density functional theory (DFT) and time-dependent DFT (TD-DFT) calculations were performed in Orca 5.0.4,^45^ while the local mode analysis was carried in LModeA^41^ software. The orange boxes describe the steps of the protocol, whereas the blue boxes express the results harnessed by the methodology.

Motivated by this endeavor, we undertake the challenging task of comprehending the role of vibronic coupling in the dynamics of ISC. In pursuit of this goal, we introduce a theoretical and computational protocol to rationalize the rates of ISC in Eu^3+^ complexes under the framework outlined by de Souza,^33^ Neese,^34^ and Barone.^35^ Our focus on these complexes was driven by their broader range of available data in the literature, which allowed us to explore the simultaneous effects of altering the ligand scaffold on the vibronic excited state processes. The methodology relies on calculating ISC rates using the correlation function (CRF) approach. Here, vibrations are considered in the Franck–Condon (FC) density of states through the S_1_ and T_1_ Hessian matrices at their respective equilibrium geometries. To ensure the suitability of the proposed method, we also compared the results with those obtained by the Marcus-Levich static approach. In addition, we further illustrate the composition of the normal vibrational modes by decomposing them into local vibrational modes using local vibrational theory.^36,37^ Specifically, the composition of normal modes (CNM) analysis,^38,39^ a special feature of our local vibrational mode analysis (LMA),^37,40,41^ was employed to fully capture the dynamics of the ISC (see Scheme 1). The obtained outcomes underscore the viability of the proposed approach and unveil new possibilities for designing novel Eu^3+^ complexes with enhanced photophysical properties.

## Results and Discussion

When aiming for an accurate description of the excited-state processes of complexes, selecting an appropriate method is crucial. One must find an affordable, i.e., low-cost computational procedure that facilitates a reliable analysis of excited-state geometries and frequencies, two factors that are often difficult to match.^42^ High-quality simulations of the excited state not only enhance the description but also provide a state-of-the-art model for Ln^3+^ complexes.^43^ To achieve this goal, the procedure summarized in Scheme 1 and detailed in Supporting Information Note S1 was employed to obtain geometries and frequencies of both S_1_ and T_1_ states for four Eu^3+^ complexes, chosen on the availability of experimental ISC rates: (a) [Eu(tta)_3_(H_2_O)_2_], (b) [Eu(tta)_4_]^−^, (c) [Eu(NO_3_)_3_(phen)_2_], and (d) [Eu(PyRCF_3_)_3_(phen)], with the following abbreviations; *tta*: 2–thenoyltrifluoroacetone, *phen*: 1,10–phenantroline, *PyrCF*_*3*_: (1-(1-methyl-1H-4-pyrazolyl)-4,4,4-trifluorobutane-1,3-dionate). The optimized molecular structures of the complexes are shown in Figure S1.

Following the geometries and frequency calculations, the vibronic parameters, including the Huang–Rhys factor and reorganization energy (λ_M_), were obtained by considering the Duschinsky rotation^44^ between the S_1_ and T_1_ states in Orca 5.0.4 (Orca_ESD module).^45^ It is worth noting that the change in geometries calculated from the Duschinsky rotation was performed using Baker’s delocalized internal coordinates,^46^ following the procedure described by Reimers.^47^ This process generated the terms required to calculate the intersystem crossing rates under the time-dependent framework. The foundation of this protocol involves adapting the computational procedure to account for the shielded nature of the 4f orbitals (detailed in the Supporting Information note S1) in the Franck–Condon density of states. The obtained properties were used in the model derived by De Souza et al.,^33^ available in Orca software. The ISC rate is then expressed as follows
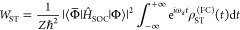
1a
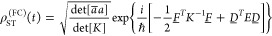
1bIn eq 1a,  represents the spin–orbit coupling matrix elements (SOCMEs) between triplet  and singlet (Φ) states, respectively, where Φ denotes the electronic part of the wave function. *Z* is the partition function, defined as , in which *E*_ν_ is the energy of the ν point of the potential energy surface (PES), while *k*_B_ stands for the Boltzmann constant, and *T* the temperature. Moreover, ℏ is the reduced Planck constant, *t* stands for time, and *ω_if_* the adiabatic energy difference. In eq 1b, *a̅* and *a* are the T_1_ and S_1_ diagonal matrices, respectively, with diagonal elements *a*_k_ and *b*_k_ dependent on the propagation time. These matrices were used to construct the *K*, *F*, and *D* matrices related to the correlation function in the Franck–Condon levels, *ρ*_ST_^(FC)^, used in this approach to accomplish the time-dependent framework. Additional details about *ρ*_ST_^(FC)^ can be found in the Supporting Information, eqs S20 and S21. It is noteworthy that the entire approach relies on normal vibrational modes, where the trace can be expressed in terms of any complete set. Thus, understanding the nature of these normal vibrations, as provided by the CNM, and identifying the local vibrational modes that dominate the vibronic coupling was a key point of our study.

**Figure 1:**
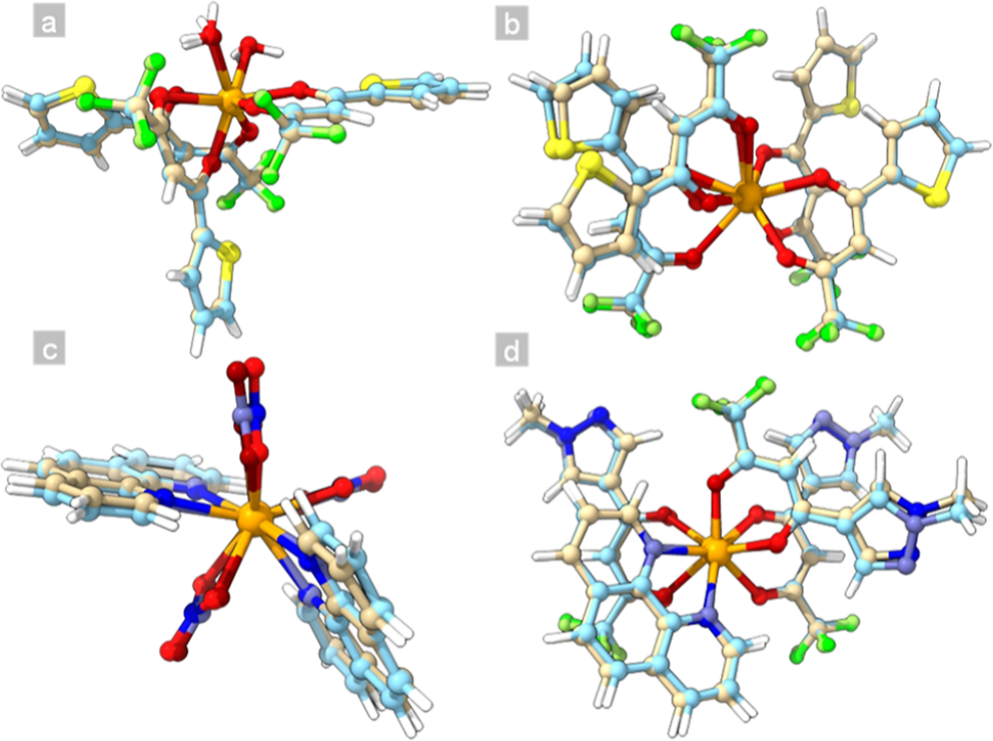
Superimposed S_1_ and T_1_ geometries optimized at the TD-DFT level of (a) [Eu(tta)_3_(H_2_O)_2_], (b) [Eu(tta)_4_]^−^, (c) [Eu(NO_3_)_3_(phen)_2_], and (d) [Eu(PyrCF_3_)_3_(phen)] complexes. The color code is adopted as follows: S_1_ geometry: blue = nitrogen, light yellow = carbon, lime green = fluorine, orange = europium, red = oxygen, yellow = sulfur, white = hydrogen. T_1_ geometry: pale blue = nitrogen, light blue = carbon, green = fluorine, orange = europium, dark red = oxygen, dark yellow = sulfur, white = hydrogen.

The optimization of both the S_1_ and T_1_ states for all the complexes revealed that each of the four complexes belongs to the C_1_ point group of symmetry for both the electronic excited states (Figure 1a–d). A discernible degree of distortion between the S_1_ and T_1_ geometries was observed and qualitatively assessed based on the root-mean-square deviation (RMSD, Table S1, calculated using eq S30). An increase in the number of atoms within the structure tends to lead to higher RMSD values, except for [Eu(NO_3_)_3_(phen)_2_], which, despite having 57 atoms, exhibited a higher RMSD (0.234 Å) than [Eu(tta)_3_(H_2_O)_2_] with 61 atoms and an RMSD of 0.155 Å. This deviation from the overall pattern can be ascribed to the relatively smaller spatial occupancy of the phen ligand compared with that of tta. Such a phenomenon allows the nitrate to adjust its positions more freely within the structure, yielding displaced Cartesian coordinates for the T_1_ state.^48^

Furthermore, the bond lengths and angles exhibited distortions from the S_1_ to T_1_ states, indicating an increase in the bond length in the first coordination sphere of Eu^3+^ for all four complexes (Tables S2–S5). The variation toward longer values stems from the greater antibonding character of S_1_, given that this state lies above T_1_ in energy. This shift in equilibrium toward greater values is a consequence of the shallower nature of the S_1_ curve in the configurational diagram.^49−51^

A comprehensive data set was obtained by comparing the structural features of the complexes. For example, an inverse trend with respect to RMSD and bond length is evident, indicating that larger ligands with greater resonance possibilities result in lower S_1_–T_1_ bond length distortions. This correlation can be further associated with the S_1_–T_1_ energy splitting,^52^ as discussed throughout this paper. Conversely, the bond angles vary in a nonlinear pattern compared with the length, being the most important factor for the RMSD considering only the atoms coordinated to Eu^3+^. Altering the structural parameters of compounds profoundly affects their electronic structures. Therefore, this understanding yields an accurate description of the structural dynamics of photophysical processes. Figure 1a enables a qualitative assessment, revealing that the positions of water ligands and thiophene rings are most affected by electronic excitation. Figures 1b–d also show that the positions of the aromatic rings change, primarily due to dihedral distortions in coordination with the Eu^3+^ ion.

After obtaining an atomic picture of the molecular distortion between the S_1_ and T_1_ states, it becomes feasible to delve into the subatomic scale of these compounds, specifically exploring their electronic structure (Supporting Information Note S4). Considering the S_1_ and T_1_ states with their potential energy surface using the correlation function approach (as illustrated in Scheme 1), the calculated ISC rates exhibited fluctuations spanning over two orders of magnitude across the set of complexes under investigation. Despite the differences in the ISC rates among each compound, our proposed protocol yielded values (Table 1, *W*_ISC-Theo_^CRF^) that were closer to experimental values than those obtained with the static Marcus-Levich approximation (Table 1, *W*_ISC-Theo_^ML^). It should be noted that due to the water vibrational modes, the [Eu(tta)_3_(H_2_O)_2_] complex remained as the exception, where both approaches yielded the same rate. This arises because of low-frequency modes, which can be treated under the ML approach at the initial state, as indicated by Marian et al.^53^

**Table 1:** Comparison of the Adiabatic S_1_–T_1_ Energy Splitting (Δ*E*_ST_), Overall Reorganization Energies (λ), and Spin–Orbit Coupling Matrix Elements () for the Four Studied Complexes as Well as Their Experimental and Theoretical Rates of Intersystem Crossing (***W***_**ISC**_)^a^

compounds	Δ***E***_**ST**_/cm^–1^	**λ**_**ML**_/cm^–1^	**λ**_**CRF**_/cm^–1^	⟨S_1_|Ĥ_SOC_|T_1_⟩/cm^–1^	***W***_**ISC**_ (theo.^ML^)/s^–1^	***W***_**ISC**_ (theo.^CRF^)/s^–1^	***W***_**ISC**_ (exp.)/s^–1^
[Eu(tta)_3_(H_2_O)_2_]	4452	3986	4116	3.34	6.6 × 10^6^	6.6 × 10^6^	2.6 × 10^7^ [^54^]
[Eu(tta)_4_]^−^	5377	4512	4482	12.7	7.1 × 10^7^	1.3 × 10^8^	4.4 × 10^8^ [^55^]
[Eu(NO_3_)_3_(phen)_2_]	4898	4148	4395	3.72	8.4 × 10^7^	4.1 × 10^8^	2.9 × 10^8^ [^56^]
[Eu(PyrCF_3_)_3_(phen)]	6107	4794	4495	12.2	8.1 × 10^9^	4.2 × 10^10^	5.7 × 10^10^ [^57^]

a The number in brackets in the experimental rates is the reference from which the values were taken. ML stands for Marcus-Levich statical approach to ISC, while CRF represents the correlation function dynamic approach in a time-dependent framework.

At first glance, we can interpret the variations in the ISC rates by analyzing the values of SOCMEs from a purely electronic standpoint, employing the El-Sayed rules (Supporting Information Note S5).^58^ According to these rules, the intersystem crossing rate significantly increases when the transition involves a change in the orbital type, thereby breaking the selection rules due to pronounced orbital mixing. In this sense, the SOCMEs for the transitions of character ^1^*n*π* → ^3^ππ* are higher than the ones for ^1^*n*π* → ^3^*n*π or vice versa for molecular compounds. For instance, organic chromophores with general transitions of character ^1^*n*π* → ^3^ππ* display ISC rates two orders of magnitude higher than their ^1^*n*π* → ^3^*n*π counterparts.^59^ Notably, the ISC rates observed in the current study fell within the 10^7^ to 10^10^ s^–1^ range, influenced in part by the principles outlined in the El-Sayed rules.

This phenomenon becomes comprehensible when we consider that the SOC operator involved in the term  is simplified as the product of the orbital and spin angular momentum (as described in eq S2). In an atomic context, the angular momentum operator rotates the atomic orbitals, altering their symmetry in real space (as illustrated in Figure S9). This rotation results in substantial spatial integrals between the initial and final states with different orbital symmetries^59^ (S_1_ and T_1_ in this study). Nevertheless, caution is warranted for the analysis of lanthanides, given that their ground and excited states are typically described by an intermediate coupling scheme.^60^ This scheme already incorporates heightened L-S mixing, which is attributable to the significant SOC originating from the 4*f* orbitals^61,62^ (see Supporting Information Note S5). Consequently, even for El-Sayed forbidden transitions (or partially forbidden for some of the cases outlined in these complexes, Table S7), the SOC matrix elements persist, maintaining the same order as the El-Sayed allowed transitions, as shown in Tables S7 and 1.

Driven by this observation, it should still be highlighted the reason for the three- to 4-fold variation in the SOCMEs within the complexes under investigation (Table 1). The main hypothesis stems from Table S7, where we notice a partially El-Sayed forbidden transition character denoted as ^1^*n*π*/^1^*n*π* →^3^*n*π*/^3^ππ and ^1^*n*π*/^1^*n*π* →^3^ππ*/^3^*n*π, for [Eu(tta)_3_(H_2_O)_2_] and [Eu(NO_3_)_3_(phen)_2_], respectively, whereas a fully allowed transition character, represented as ^1^*n*π*/^1^*n*π* *→*^3^ππ*/^3^ππ, is observed (Table S7) for [Eu(tta)_4_]^−^, and [Eu(PyrCF_3_)_3_(phen)]. Nonetheless, despite this disparity, both transitions exhibit the same order of magnitude owing to the aforementioned L–S mixing effect. Another aspect potentially contributing to the non-null SOCMEs for the El-Sayed partially forbidden transition lies in the dependence of the SOC on the geometry, particularly the distance between the ligand centroid orbitals and the lanthanide ion. Consequently, shortening this distance tends to amplify SOCMEs due to enhanced orbital overlap.^63^

Despite the variations in the SOCMEs, their quadratic dependence alone is insufficient to account for the differences in the magnitude of ISC from [Eu(tta)_3_(H_2_O)_2_] to [Eu(PyrCF_3_)_3_(phen)], as it exclusively considers electronic effects. Therefore, two additional features deserve further analysis: (*i*) the S_1_–T_1_ energy splitting (Δ*E*_ST_ in Table 1) and (*ii*) the vibrational density of states (VDOS). Concerning the former, the existing literature commonly associates bulkier ligands with multiple aromatic rings to lower T_1_ state energy of the complex.^64^ However, as exhibited in Table 1, an inverse correlation is evident: with an increase in ligand bulkiness, the complex exhibits higher energy gaps between the S_1_ and T_1_ states. This trend persisted throughout the entire series under investigation. Notably, although the [Eu(PyrCF_3_)_3_(phen)] complex has the highest energy gap, it exhibits a greater ISC across the series (Table 1). This observation underscores the role played by the VDOS in controlling the rates.

A direct correlation is evident between the S_1_–T_1_ energy splitting (Δ*E*_ST_) and reorganization energy (λ), as depicted in Table 1. The latter reflects the energy needed to readjust the equilibrium configuration from the S_1_ state to the T_1_ state, with higher values corresponding to larger energy gaps, which agrees with the anticipated expectations. These values can be further correlated with the degree of distortion between the two geometries, as discussed in the structural analysis. These observations indicate that the S_1_–T_1_ energy splitting is proportional to the system rigidity, which is similar to the behavior of thermally activated delayed fluorescence (TADF) emitters.^48,65^ This is an elegant outcome from this analysis, as from a pure electronic perspective, to enhance the population of T_1_ and therefore augment the energy transfer to Ln^3+^, one can picture a more rigid complex due to the lowering of Δ*E*_ST_ at a reasonable amount, avoiding reverse intersystem crossing.^66^ In addition, this result agrees with the Franck–Condon (FC) approximation to the VDOS, which was used in this study because of the reasonable SOCMEs (>3 cm^–1^) that inhibit the necessity to include the Herzberg–Teller (HT) effect.^35^

An interesting observation arises when reorganization energies are incorporated into the determination of ISC. In the Marcus-Levich (ML) statical approach, both the reorganization energies and the S_1_–T_1_ energy difference (designated as λ_ML_ and Δ*E*_ST_, respectively) are directly factored into the exponential component of the eq (eq S1). In contrast, in this methodology (CRF), the FC factors for the rates account for the contribution from the normal vibrational modes to the reorganization energy.

While both methods (ML and CRF) aim to describe the reorganization energy (λ_ML_ and λ_CRF_, respectively), their theoretical bases may lead to distinct values. The ML approach incorporates nuclear displacements through a semiclassical perspective, where the initial-state nuclear distributions are treated classically, and the dynamics in the final state are not explicitly considered. As a result, ML effectively reduces all vibronic effects to a single parameter (λ_ML_), which can be a reasonable approximation if low-frequency modes dominate the nuclear relaxation process, being a computationally cost-effective method. However, this approach does not explicitly incorporate the full vibrational structure of the system, which may lead to divergences when comparing with the λ_CRF_ values.

On the other hand, the CRF approach explicitly accounts for vibronic coupling within the harmonic approximation, offering a more detailed description of the nuclear relaxation process in the final state. Nevertheless, it does not inherently make λ_CRF_ superior to λ_ML_, as the agreement between the two approaches holds only under specific conditions—namely, when the PES of the initial and final states are both perfectly harmonic and share the same curvature. Even when these conditions are met, differences may arise due to anharmonic effects, which are not captured by the λ_CRF_. Therefore, rather than considering one approach as generally superior, the choice between ML and CRF should be made based on the specific characteristics of the system under investigation, balancing computational efficiency and theoretical rigor.

In summary, vibronic details are incorporated within the CRF approach by considering the difference between points in each PES, whereas in the ML approach, the overall effect is considered.

**Figure 2:**
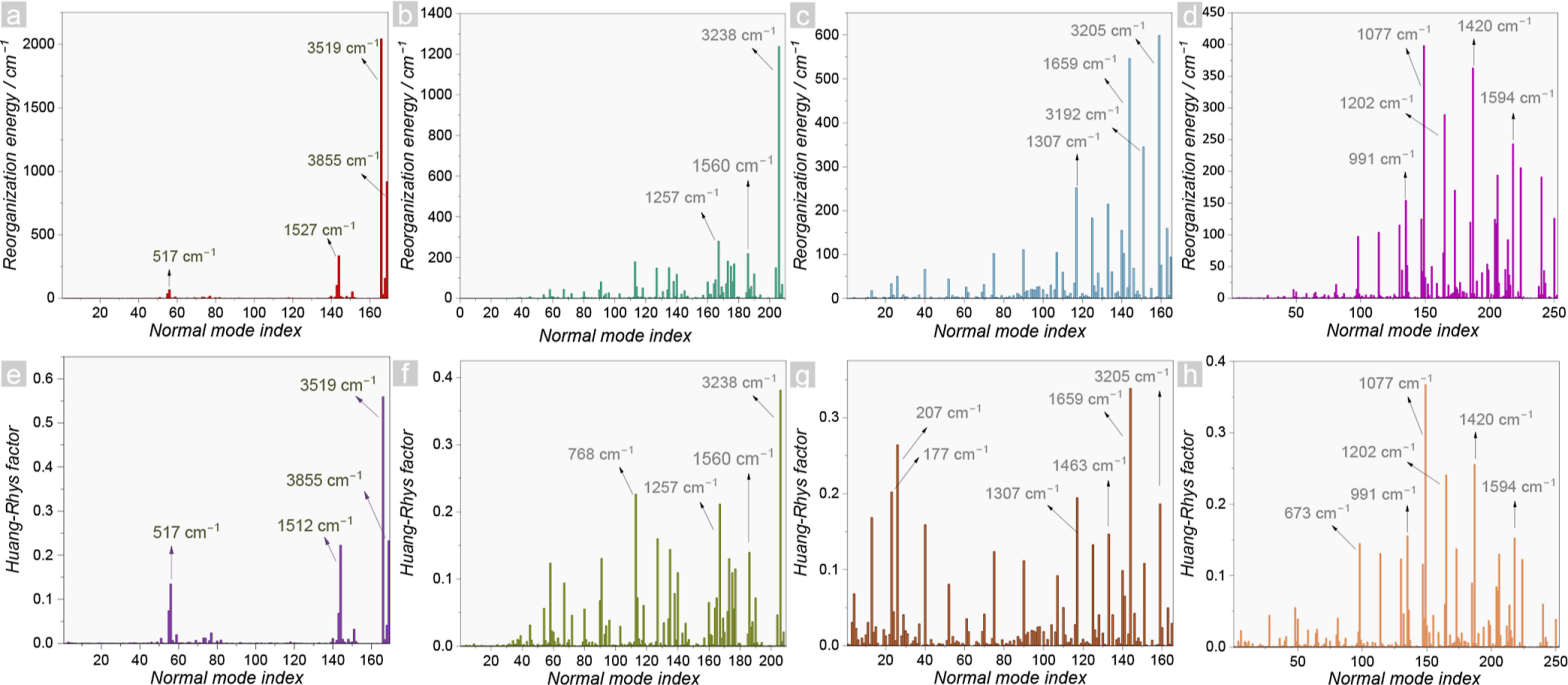
Reorganization energies per normal vibrational modes for (a) [Eu(tta)_3_(H_2_O)_2_], (b) [Eu(tta)_4_]^−^, (c) [Eu(NO_3_)_3_(phen)_2_], (d) [Eu(PyrCF_3_)_3_(phen)] in wavenumber and dimensionless Huang–Rhys factor per vibrational modes for (e) [Eu(tta)_3_(H_2_O)_2_], (f) [Eu(tta)_4_]^−^, (g) [Eu(NO_3_)_3_(phen)_2_], (h) [Eu(PyrCF_3_)_3_(phen)]. Only the wavenumber of the normal modes with the higher contribution for each complex are highlighted for the sake of clarity.

**Figure 3:**
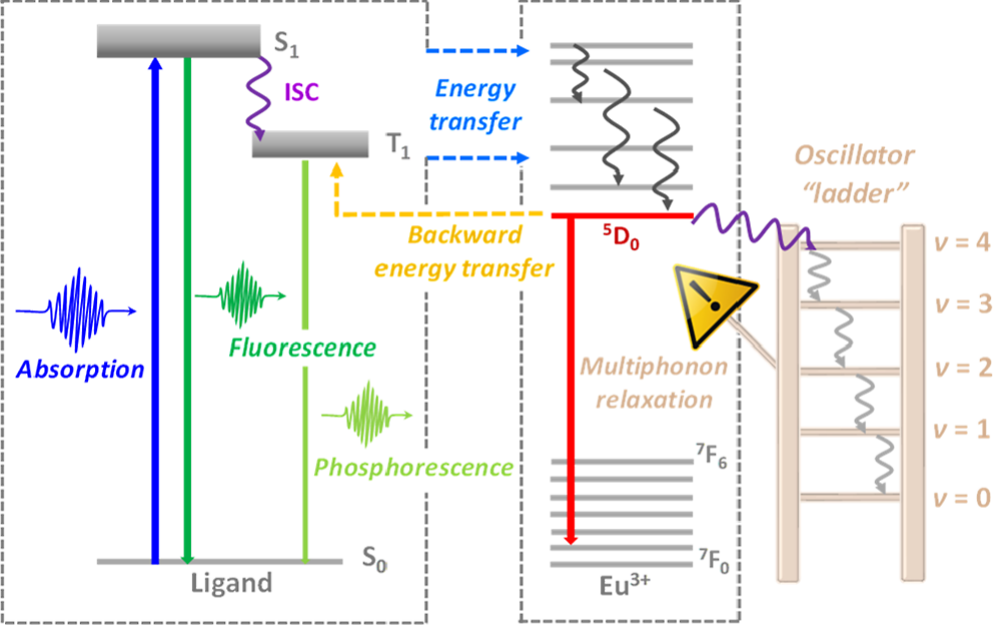
Multiphonon relaxation scheme in an Eu^3+^ complex where the oscillator ladder can be seen as the acceptor vibrational mode.

It is worth noting that the reorganization energy varies according to the displacement of the S_1_–T_1_ configurational diagram, and therefore, with each normal vibrational mode, leading us to Figure 2a–d. Concomitantly, using eq S35, the Huang–Rhys (HR) factor per vibrational mode is obtained, which quantifies the electron-vibrational coupling (Figure 2e–h).^67,68^ The complete values for the four complexes are highlighted in Tables S8–S11 (for further information regarding the vibronic part, see Supporting Information Note S6). From Figure 2a,e, we notice that water vibrations in the [Eu(tta)_3_(H_2_O)_2_] complex, more specifically, the O–H stretching, are primarily responsible for the vibronic coupling. Because of small contributions of other fragments, the vibronic coupling is dominated by low-frequency modes, making the ML approach also appropriate for this complex. The O–H oscillations are also responsible for deactivating the ^5^D_0_ excited state of Eu^3+^ via multiphonon relaxation^69,70^ (schematized in Figure 3 as the oscillator ladder). As mentioned in the introduction, the ^5^D_0_ state is populated via energy transfer from ligand states, whereas the sequential emission of vibration quanta (oscillator ladder induced by multiphonon relaxation) is the main pathway to deactivate this state.^53^ This leads to lower emission quantum yield compared to complexes where O–H fragments are absent.^71^

One strategy to overcome this deactivation is to replace water molecules with ligands possessing lower vibrational energies,^64^ such as those in the [Eu(tta)_4_]^−^ complex. Within this approach, it is notable that the reorganization energy is maintained in high-energy oscillations, however, stemming from C–H stretching (3238 cm^–1^) in this case. In contrast, the Huang–Rhys factor is more distributed across normal modes, implying that modes with intermediate frequencies (700–1600 cm^–1^) contribute effectively to vibronic coupling. This modification directly affected the ISC rates, as an increase of 1.5-fold (8.4 × 10^7^ to 1.3 × 10^8^ s^–1^, Table 1) was observed when the coupling was maintained with less energetic modes.

An additional resource at our disposal for the analysis is the Duschinsky rotation matrix (Figure S11). For the [Eu(tta)_3_(H_2_O)_2_] complex (Figure S11a), the deviation from the diagonality indicates a high mode mixing index between the two electronic states (S_1_ and T_1_).^72^ This observation suggests that the presence of water molecules coordinated to the Eu^3+^ ion amplify mode mixing compared to the [Eu(tta)_4_]^−^ complex, for example, resulting in disordered coupling between the vibrational modes. Such disordered coupling could hinder further interactions between the electronic and vibrational states, restricting the HR factor to a more localized character.^73^

From these two examples, one can conclude that replacing water fragments effectively enhances the ISC rates involved. However, it prompts the question of whether this behavior persists across the series. This inquiry can be addressed by directly examining the [Eu(NO_3_)_3_(phen)_2_] and [Eu(PyrCF_3_)_3_(phen)] complexes. In these complexes, items c, d, and g, h in Figure 2 exhibit a more evenly distributed Huang–Rhys factor and reorganization energies across the normal vibrational modes. In the [Eu(NO_3_)_3_(phen)_2_] complex, a spreading of normal modes is observed (Figure 2g), effectively contributing to vibronic coupling. In addition, the reorganization energy (Figure 2c) was greater for high-energy oscillations due to wavenumber weighting, potentially quenching the Eu^3+^ luminescence. Conversely, the [Eu(PyrCF_3_)_3_(phen)] complex displays higher vibronic coupling, as indicated by the reorganization energies in Table 1. However, it is noteworthy that only modes with intermediate frequencies (600–1600 cm^–1^) contribute efficiently. This characteristic may confer an advantage on the luminescence of the complex because it likely prevents multiphonon relaxation. By analyzing the Duschinsky matrix of both complexes (Figure S11c,d), it becomes apparent that the [Eu(PyrCF_3_)_3_(phen)] complex exhibits lower S_1_–T_1_ mode mixing than [Eu(NO_3_)_3_(phen)_2_], as evidenced by its higher diagonality index. Such result aligns with previous observations that lower mode-mixing enhances the ISC rates by allowing more distributed vibronic coupling.

To further broaden the scope of the analysis provided by the Duschinsky matrix, it is important to note that all complexes are assigned to the *C*_1_ point group. In this group, the rotations share the same symmetry representation (A) as the displacements stimulated by normal vibrations. This sets the stage for an axis-switching effect,^74^ which disrupts the linearity of the Duschinsky matrix, potentially resulting in nondiagonal elements even in the absence of mode mixing. Consequently, in this study, the emphasis is not solely on the absolute values of diagonality but rather on the overall form of the matrix.^74^

A direct interpretation of the vibrational density of states is not trivial for such complexes because of the large number of atoms and the presence of a metal center.^75^ Under these circumstances, one can resort to a correlation function to comprehend the dynamics of the VDOS (Figure S12).^76,77^ The form of the function oscillates with respect to time, except for the [Eu(NO_3_)_3_(phen)_2_] complex where longer convergence periods is necessary, revealing the static structure of VDOS for this compound. This indicates that although static approaches are adequate for describing this complex, a short-time approximation cannot be employed,^78^ unlike for the other three complexes, where shorter times are required for function convergence. In these cases, large oscillations were observed (Figure S12a,d).

Many of our promising findings hinge on the normal vibrational modes that influence vibronic coupling. The discussion covers energy ranges from high stretching energies (>3000 cm^–1^) to intermediate energies (700–1600 cm^–1^), encompassing a vast number of vibrations. However, in polyatomic systems, the nature of normal vibrations is characterized by their delocalization throughout the molecule in the form of collective fragment motions (Figures S13–S16).^40^

**Figure 4:**
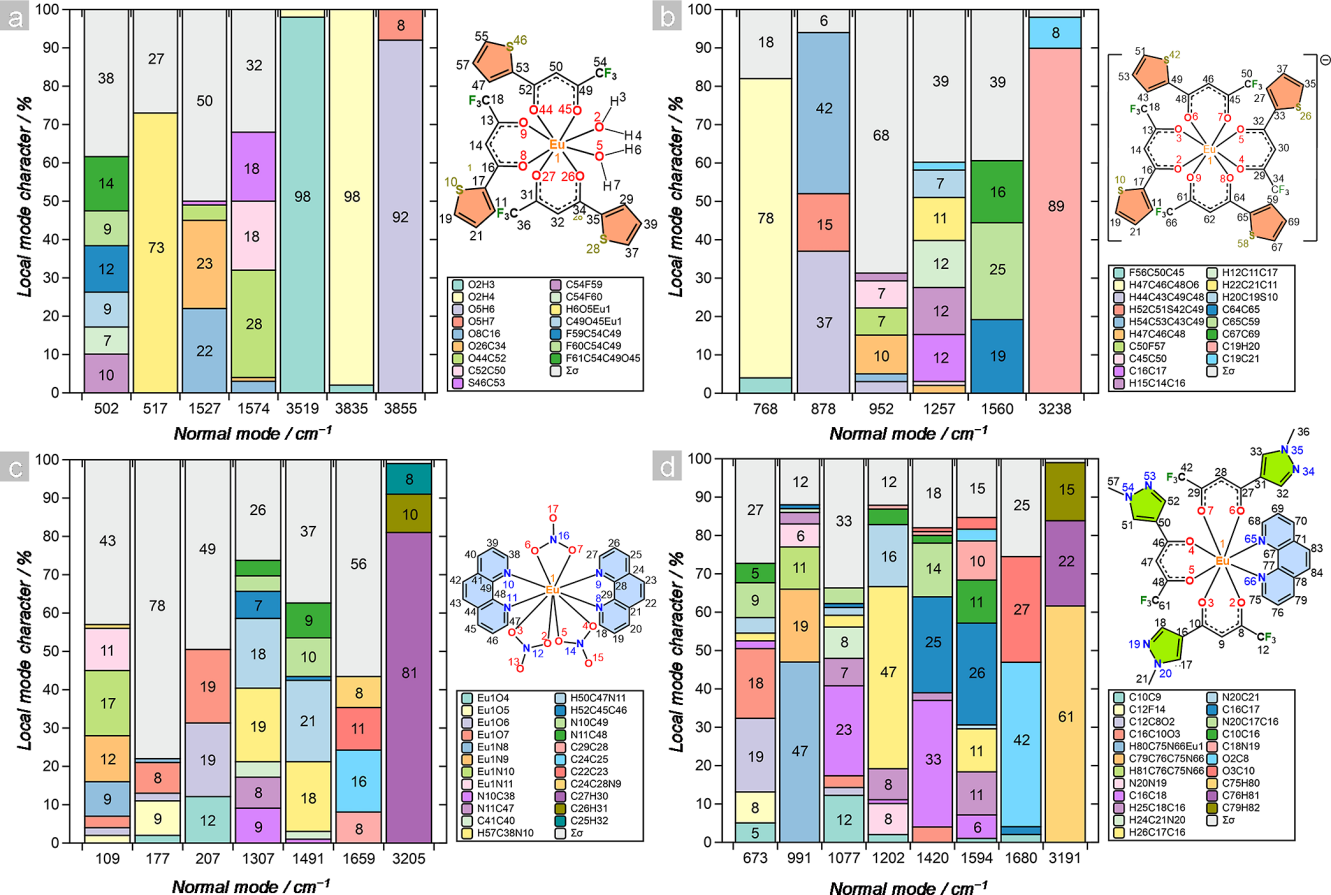
Decomposition of normal modes into local mode contributions via the CNM analysis for the vibrations that contribute most to the Huang–Rhys factor and reorganization energy presented in Figure 2 for (a) [Eu(tta)_3_(H_2_O)_2_], (b) [Eu(tta)_4_]^−^, (c) [Eu(NO_3_)_3_(phen)_2_] and (d) [Eu(PyrCF_3_)_3_(phen)] complexes. ∑*σ* represents the sum of all the local mode contributions from the remaining fragments.

To quantitatively analyze the contributions of the ligand scaffold fragments, decomposing the normal vibrational modes into local vibrational mode (LVM) contributions^79−82^ is imperative. This process is based on the 1:1 relationship between the (3*N*-6) normal vibrational modes (with *N* being the number of atoms) and a complete nonredundant set of (3*N*-6) local vibrational modes, known as the adiabatic connection scheme (ACS).^83^ The ACS lays the foundations for the composition of normal mode (CNM) analysis.^36^ In this context, by selecting the six to eight most influential oscillations that contribute to the reorganization energies and Huang–Rhys factors (Figure 2) and decomposing them into LVMs, we provide Figure 4. This figure summarizes the weighted contributions of each molecule fragment. Throughout the CNM analysis, it is noticed that the high-energy oscillations in [Eu(tta)_3_(H_2_O)_2_] arose completely from the water molecules in the coordination sphere (Figure 4a). However, the normal modes at 517 and 1527 cm^–1^ contribute to the vibronic coupling (Figure 2e) with a minor share, and these modes exhibit a more delocalized character, as they are composed of several molecular fragments (Figure 4a).

The substitution of water molecules by the tta^–^ ligand (i.e., in [Eu(tta)_4_]^−^) redistributes the vibronic coupling, increasing the involvement of other vibrational modes, as previously mentioned (Figure 2f). However, the high-energy oscillations maintained a predominantly local character (approximately 97%) of C–H stretching, while higher delocalization is observed in the intermediate energy region (700–1600 cm^–1^), except for the normal mode vibration at 768 cm^–1^. This specific normal mode comprises 78% of the H–C–C–O dihedral angle distortion between carbons and oxygen in the tta^–^ ligand (details in Figure 4b). Interestingly, the Eu^3+^ ion does not participate in the relevant normal modes for vibronic coupling in [Eu(tta)_4_]^−^, whereas it contributes to the Eu–O–H deformation (73%) at 517 cm^–1^ in the [Eu(tta)_3_(H_2_O)_2_] complex (Figure 4a).

A similar trend is observed for the [Eu(NO_3_)_3_(phen)_2_] complex, where the highest reorganization energy stems from the 3209 cm^–1^ normal vibrational mode associated with the C–H stretching (contribution of 81%, Figure 4c). However, the highest Huang–Rhys factor does not correspond to the reorganization energy; instead, it originates from the vibration at 1659 cm^–1^ (Figure 2c,g). Consequently, when solely considering the Huang–Rhys factor, which defines the overlap of vibrational wave functions,^68^ a highly mixed nature of the 1659 cm^–1^ normal mode is observed. This mode encompasses several contributions, including torsional motion in the aromatic rings of the phen ligand (Σ*σ*) as well as C=C stretching. Furthermore, this compound presents three low-energy modes with high Huang–Rhys factor S, characterized by contributions from the Eu–O and Eu–N bonds, except for the 177 cm^–1^ mode, where contributions from torsion of the aromatic rings and interconversion between the nitrate accounts for 78% of the vibrational mode.

The complex exhibiting the highest intersystem crossing rate, i.e., [Eu(PyrCF_3_)_3_(phen)], showcased the most pronounced normal mode delocalization across various regions of the molecule (Figure 4d). Even the vibration with the highest energy (3191 cm^–1^) has a lower contribution from a single stretch (61%) than the others. However, this mode contributes minimally to vibronic coupling. Therefore, upon conducting a more intricate analysis of the normal modes in the intermediate energy region, it becomes apparent that this ensemble of vibrations manifests a highly delocalized character (Figure 4d). To tackle this delocalization, we focus on normal modes with similar fragments. For instance, both the 1077 and 1420 cm^–1^ modes comprise C=C stretching on the heterocyclic ring of the PyrCF_3_ ligand (23% and 33%, respectively). The primary distinction between these modes lies in the significant contribution of the adjacent C=C stretching for the 1420 cm^–1^ normal mode (25%), which is absent for the normal mode with frequency at 1077 cm^–1^. Conversely, the normal vibrational mode associated with the energy between these two (1202 cm^–1^) exhibits the most localized character within the energy region of interest, with a share of 47% attributed to the C=C–H deformation in the heterocyclic ring of PyrCF_3_.

The three-dimensional structure reveals that this ring is distorted in the out-of-plane direction for both oxygens of the ligand (Figure S16). This distortion enhances the possibility of interactions with hydrogen by reducing the distance between oxygen and hydrogen in the rings.^50^ Overall, the absence of high-energy oscillations actively engaging in vibronic coupling in these complexes constrains potential multiphonon relaxation. Meanwhile, the involvement of multiple molecular fragments in the formation of the most relevant normal modes for coupling enhances the vibrational density of states and, consequently, intersystem crossing rate.

The successful elucidation of the role of vibronic coupling in the intersystem crossing between S_1_ and T_1_ states, along with the attainment of a desirable agreement between experimental and theoretical rates, underscores the potential of the proposed methodology in describing the excited-state processes of Eu^3+^-based complexes. In addition, CNM has unveiled the composition of the vibrations most relevant to vibronic coupling, offering insights into the molecular fragments that constitute each oscillation. By combining this approach with the decomposition of normal modes into the contributions of local vibrations, we can customize the ligand scaffold toward brighter Eu^3+^ compounds by reducing the energies of the most relevant vibrations for vibronic coupling. These adjustments present a promising strategy for enhancing the quantum yield as well as designing new materials for photonics.

## Conclusion

This study delved into the excited-state processes of Eu^3+^-based coordination compounds using a systematic theoretical procedure for estimations of S_1_-T_1_ intersystem crossing rate (ISC). The primary focus of this study was to reconcile theoretical predictions with experimental observations regarding the ISC rates, which were achieved by considering the vibronic coupling effects originating from the vibrational density of states. The accuracy of this protocol is a direct consequence of using the difference between points in the S_1_ and T_1_ potential energy surfaces, allowing the inclusion of detailed vibronic effects. A notable finding is the correlation between lowering the mixing of S_1_–T_1_ modes and enhancing the involvement of vibrations within the 700–1700 cm^–1^ energy range in vibronic coupling. This correlation hints at the potential for increased populations of the T_1_ state solely through ISC, challenging the conventional notion of the unfavorable nature of vibronic coupling in Ln^3+^-based complexes. It was observed that unfavorable outcomes predominantly stemmed from the contributions of high-energy oscillations (>3000 cm^–1^), which resulted in the effective deactivation of the ^5^D_0_ manifold of Eu^3+^ via multiphonon relaxation. Furthermore, a novel protocol was introduced to leverage enhanced photophysical properties across various compounds, focusing primarily on Eu^3+^ complexes. By analyzing vibrations corresponding to each molecular fragment within the referenced normal mode, pathways for tailoring the ligand scaffold to either diminish the energy of oscillations or increase its participation in vibronic coupling were elucidated. Future studies will focus on ISC in these compounds using nonadiabatic approaches as well as expanding to the vertical Hessians framework. Such works hold promises for new insights and refining the understanding and modeling of excited-state processes, potentially increasing the accuracy of current methods. The strategy introduced in this study marks a departure from the conventional analysis of ISC, which is frequently employed to model complexes and project novel compounds.

## Supporting Information Available

Further details of the theoretical methodology developed for this study, including all results harnessed by the computational analysis and the values used to compute the rates. The structure of all complexes in each excited state is also shown, highlighting the distortion between the geometries in the singlet and triplet excited states. Vibrational analysis obtained by computing the numerical Hessians and subsequent local mode analysis is also shown (PDF)

## Abbreviations

ISC: intersystem crossing
ET: energy transfer
IET: intermolecular energy transfer
ML: Marcus-Levich
CRF: correlation function
CNM: composition of normal modes
LMA: local mode analysis
LVM: local vibrational modes.
