# Supplementary material for: Role of Vibronic Coupling for the Dynamics of Intersystem Crossing in Eu3+ Complexes: an Avenue for Brighter Compounds
Source: J Chem Theory Comput. 2025 Mar 7;21(6):3066–76. doi: 10.1021/acs.jctc.4c01461 (PMC11948322; doi:10.1021/acs.jctc.4c01461)
Supplement: Supplementary file 1 — ct4c01461_si_001.pdf [file ct4c01461_si_001.pdf]

Electronic supplementary information

The role of vibronic coupling for the dynamics of intersystem crossing in Eu^{3+} complexes: An avenue for brighter compounds

Leonardo F. Saraiva, Albano N. C. Neto, Airton G. Bispo Jr, M. Quintano, E. Kraka,* Luís D. Carlos, Sergio A. M. Lima, Ana M. Pires,* Renaldo T. Moura Jr.*

Table of contents

Supplementary note S1 – Computational procedure .. 2
Electronic structure calculations 2
Rates of Intersystem crossing 2
Local vibrational mode analysis 3
Figure S1 3
Supplementary note S2 – Theoretical aspects............ 3
Spin-orbit coupling and matrix elements................ 3
Intersystem crossing 5
Local vibrational analysis 9
Supplementary note S3 – Structural analysis 9
Table S1 10
Table S2 10
Table S3 10
Table S4 11
Table S5 11
Figure S4 12
Supplementary Note S4 – Electronic structure.......... 13
Table S6 13
Figure S5 13
Table S7 15
Supplementary Note S5 - El-Sayed rules for intersystem crossing and electronic structure of lanthanides 16
Figure S10 17
Supplementary Note S6 – Vibronic properties........... 19
Table S8 19
Table S9 23
Table S10 28
Table S11 32
Figure S13 40
Figure S14 40
Figure S15 41
Figure S16 42
References.. 43

Supplementary note S1 – Computational procedure

Electronic structure calculations

All electronic structure calculations were conducted in Orca 5.0.4 software package[1]. The adopted procedure encompassed an initial optimization of the ground state (S_0) geometry within the framework of density functional theory (DFT) employing B3LYP functional[2] alongside Grimme's D4 dispersion correction[3] (B3LYP-D4) for all complexes under study (Fig S1). During the optimization, all atoms except Eu^{3+} were treated with the all-electron triple−ζ Def2−TZVP basis set[4], while the adapted Stuttgart-Cologne MWB52 effective core potential[5,6] was applied for Eu^{3+}. The optimized geometry was assured by the absence of imaginary frequencies in the computation of the ground state hessian. After the S_0 optimization, the geometry of the first singlet excited state (S_1) was optimized using time-dependent density functional theory approach (TD-DFT)[7] employing the B3LYP-D4/Def2-TZVP(MWB52) procedure. Furthermore, the geometry of the first triplet state (T_1) was optimized with the same approach used for the S_0 state. After this initial step, a numerical hessian calculation using the ωB97X−D4 range separated hybrid functional[8] was performed for S_1 and T_1 states using their respective optimized geometry to extract the normal vibrational modes.

Simultaneously, the same functional (ωB97X−D4) was used to harness the spin-orbit coupling matrix elements (SOCMe) from an excited-state TD-DFT calculation[9]. However, in this step we have employed the scalar relativistic Douglas-Kroll-Hess approximation[10] at second order (DKH2) with the specifically tailored scalar relativistic recontracted SARC2-QZVP basis set[11] for Eu^{3+}. It is worth noting that the multiplets were mixed via the spin-orbit mean field (SOMF) operator[12]. Subsequent to this determination, an input file for the *Orca_ESD* (excited-state dynamics) was constructed upon the S_1 geometry, containing the S_1 and T_1 hessian matrices and the SOCMEs for the calculation of the ISC under the correlation function framework.

Rates of Intersystem crossing

The determination of the intersystem crossing (ISC) rates can be summarized in two steps: (*i*) obtention of the reorganization energy (λ_i) for each normal mode and Huang-Rhys factors (S_i) using the calculated S_1 and T_1 Hessian matrices in their optimized minima in the *Orca_ESD* module[13,14]. (*ii*) Application of the time-dependent approximation under the quantum electrodynamics framework to avoid estimated values of the vibrational density of states (VDOS)[15]. For comparison means, the rates of ISC were also determined by the Marcus-Levich statical framework (Eq. S1) [16,17,18,19]. It is worth highlighting that for this approach the total reorganization energy (λ_M) was calculated through the method reported in reference [20]. The method consists in approximating the reorganization energy using the energies of vertical excitations ($S_0 \rightarrow S_1$ and $S_0 \rightarrow T_1$) determined from the TD-DFT calculation and the adiabatic minima of S_1 and T_1, obtained from the geometry optimization (Eq. S2). It is important to note that because of the approximation, the reorganization energies employed in the Marcus-Levich method may present different values than those used in the correlation function approach. In this sense, this comparison is advantageous for the study, as it allows us to analyze how a more complete description performs in describing the excited-state processes of Ln^{3+} complexes.

$$W_{ISC} = \frac{2\pi}{\hbar} \langle S_1 | \hat{H}_{SOC} | T_1 \rangle^2 \frac{1}{\sqrt{4\pi\lambda_M k_B T}} e^{-\left[\frac{(\Delta E_{ST}+\lambda_M)^2}{4\lambda_M k_B T}\right]} \quad \text{(S1)}$$

$$\lambda_M = \frac{1}{2}[(E_{T1vertical} - E_{S1adiabatic}) + (E_{S1vertical} - E_{T1adiabatic})] \quad \text{(S2)}$$

Local vibrational mode analysis

The normal vibrational modes that most contribute to the reorganization energy and the Huang-Rhys factor were analyzed by their decomposition into local vibrational modes, which is an efficient process as described in several references [21,22,23]. This procedure was performed from the automated generation of the non-redundant and complete sets of local vibrational modes using the newly developed *LModeAGen* protocol.[24] The corresponding local mode analysis was performed with the standalone *LModeA* package[25] using the first singlet excited-state Hessian. The present step aimed to elucidate the composition of normal modes to gain insights into the fragments that enable vibronic coupling.

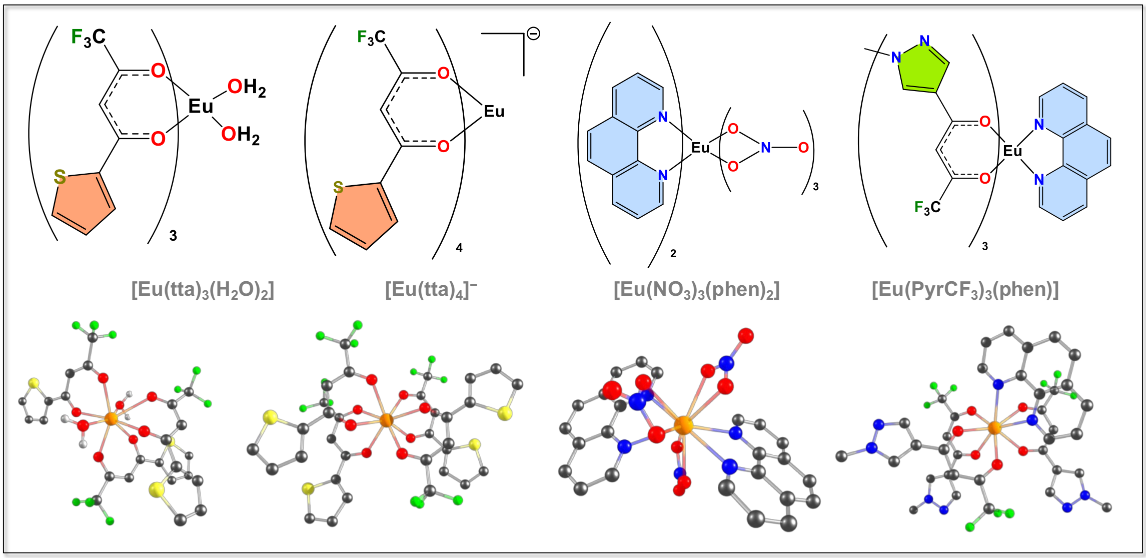

Figure S1. Structures of the Eu^{3+} complexes under study. Blue = nitrogen, green = fluorine, grey = carbon, orange = europium, red = oxygen, yellow = sulfur.

Supplementary note S2 – Theoretical aspects

In this section, some important theoretical aspects to the determination of ISC rates and vibrational mode analysis are highlighted. One should note that the theory behind this process was well-elucidated previously, and this brief revisit is necessary for more comprehensive interpretations under the framework of Neese,[12] Marian,[26] De Souza,[13,14] Pollak,[27] Shuai,[28] Peng,[29] and Barone[30].

Spin-orbit coupling and matrix elements

When increasing the atomic number of the elements involved, the spin-orbit coupling becomes non-negligible, and is considered as an interaction between the electron's spin and its orbital motion around the nucleus[31]. When an electron moves in the finite electric field of the nucleus, the spin-orbit coupling causes a shift in the atomic energy levels as consequence of an electromagnetic interaction between the spin and the electric field. While occurring this phenomenon a magnetic field generated by the interaction of the angular momentum of the electron and the electric field of the nucleus is created. The general derivation of spin-orbit coupling from the Dirac equation for an electron of mass *m* and charge −*e* is depicted in Eq. S3, where *c* is the speed of light, $\hbar$

is the Planck's reduced constant, $E(\vec{r})$ is an external electric field ($E(\vec{r}) = \nabla\phi(r)$), and $\vec{P}$ is the momentum operator while $\hat{\sigma}$ is the Pauli spin matrices.

$$H_{SO} = \frac{e\hbar}{4m^2c^2}\hat{\sigma}\cdot\left[E(\vec{r})\times\vec{P}\right] \tag{S3}$$

In this work we employed the spin-orbit mean field (SOMF) operator that is analogous to the treatment of the electron-electron interaction in the Hartree-Fock method. In the SOMF formalism, the SOC operator has the appearance of a quasi-one-particle operator (Eq. S4) where the matrix elements of $\hat{z}(i)$ over a basis set $\{\varphi\}$ is written in Eq. S5, where ρ is the electron density. The possibilities of spin-orbit coupling between singlets and triplets are illustrated in Figure S2.

$$\hat{H}_{SOMF} = \sum_i \hat{z}(i)\hat{s}(i) \tag{S4}$$

$$\langle\varphi_u|\hat{z}|\varphi_v\rangle = \langle\varphi_u|\hat{h}^{SOC}|\varphi_v\rangle + (\varphi_u\varphi_v|\hat{g}^{SOC}|\rho) - \frac{3}{2\sum_{\kappa\mathrm{T}}P_{\kappa\mathrm{T}}\left[(\varphi_\mu\varphi_\kappa|\hat{g}^{SOC}|\varphi_T\varphi_v) + (\varphi_T\varphi_v|\hat{g}^{SOC}|\varphi_\mu\varphi_\kappa)\right]} \tag{S5}$$

The manageable expression for the matrix elements lying on the SOMF operator can be obtained by using the second quantization formalism. The one-electron SOC operator in the second quantization approach is denoted in Eq. S6. In this form, the term $\frac{1}{2}\left[\hat{a}_p^+\hat{a}_q - \hat{b}_p^+\hat{b}_q\right]$ corresponds to the spin density operator, while $-\frac{1}{\sqrt{2}}\hat{a}_p^+\hat{b}_q$ and $\frac{1}{\sqrt{2}}\hat{b}_p^+\hat{a}_q$ are the spin-raising and spin-lowering operators.

$$\hat{H}_{SOMF} = \frac{1}{2}\sum_{pq} z_{pq}^-\hat{a}_p^+\hat{b}_q + z_{pq}^+\hat{b}_p^+\hat{a}_q + z_{pq}^0\left[\hat{a}_p^+\hat{a}_q - \hat{b}_p^+\hat{b}_q\right] \tag{S6}$$

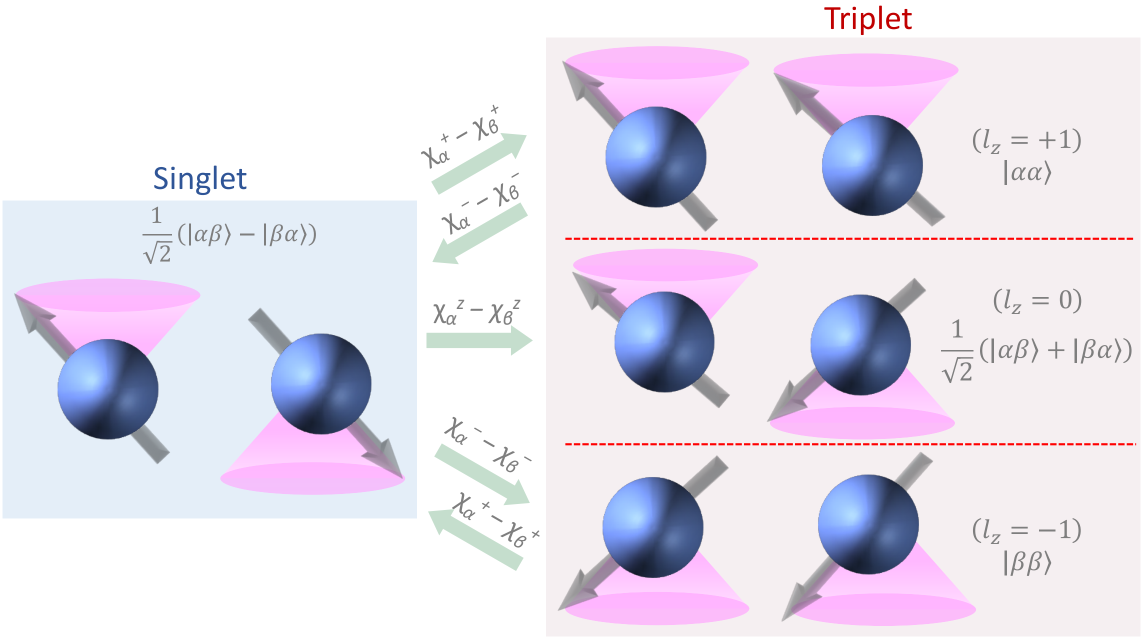

Figure S2. Schematics of singlet and triplet states of two identical spin ½ reproducing the

possibilities of spin-orbit coupling. I_z represents the z-component of the total nuclear spin. The wavefunction of the singlet state is antisymmetric and those of the triplet states are symmetric with respect to the permutation of two identical nuclei. The antisymmetric nuclear-spin operator $i_a - i_b$, where i_a and i_b are the nuclear-spin operators couples the singlet with the triplet state. $i^{\pm}_{a,b}$ represents creation and annihilation operators. In the case of multi electrons spins, the antisymmetric electron spin operator couples the electron-spin state with the triplet state.

Intersystem crossing

If the coupling between the two states of interest (S_1 and T_1) is significantly weaker compared to their adiabatic energy difference, the population transfer rate (*W*) can be approximated through perturbation theory, specifically the Fermi's golden rule (Eq. S7)[26]. Hereafter, the final state (T_1) will be assigned with a bar symbol over the term. In this equation, $\overline{\Psi}$ and Ψ are the total molecular wavefunction (electronic and vibrational components) of the triplet and singlet states, respectively. The Hamiltonian designated to this process ($\widehat{H}_{S_1\rightarrow T_1}$) describes the coupling between these states[26]. Considering the adiabatic Born-Oppenheimer and spin-orbit coupling as the interacting Hamiltonians ($\widehat{H}_{BO}$, $\widehat{H}_{SOC}$, respectively) specified in Eq. S8, the rates from the initial to final states ($W_{S_1\rightarrow T_1}$, abbreviated as W_{ST}) are expressed in Eq. S9, where $e^{-\frac{E_v}{k_BT}}$ (abbreviated as Z) is the partition function. The wavefunctions are further decomposed into their respective vibrational and electronic parts regarding the Hamiltonian (Eq S10). The spin-orbit coupling matrix elements considering the Herzberg-Teller (HT) limit and using $Q_0 = 0$ as the reference configuration yield Eq. S11. The Born-Oppenheimer counterpart is introduced in Eq. S12, where the partial derivative of the vibrational wavefunction is considered as the operator $\bar{\widehat{P}}_k$.

$$W_{ST} = \frac{2\pi}{\hbar}\sum\left|\langle\overline{\Psi}|\widehat{H}_{S_1\rightarrow T_1}|\Psi\rangle\right|^2\delta(E-\bar{E}) \tag{S7}$$

$$\widehat{H}_{S_1\rightarrow T_1} = \widehat{H}_{BO} + \widehat{H}_{SOC} \tag{S8}$$

$$W_{ST} = \frac{2\pi}{\hbar}\sum e^{-\frac{E_v}{k_BT}}\left|\langle\overline{\Psi}|\widehat{H}_{BO} + \widehat{H}_{SOC}|\Psi\rangle\right|^2\delta(E-\bar{E}) \tag{S9}$$

$$\widehat{H}_{S_1\rightarrow T_1}\Psi_v = \widehat{H}_{BO}\Phi(r)\Theta_v(Q) + \widehat{H}_{SOC}\Phi(r)\Theta_v(Q) \tag{S10}$$

$$\begin{aligned}\langle\bar{\Phi}\bar{\Theta}_u|\widehat{H}_{SOC}|\Phi\Theta_v\rangle &= \langle\bar{\Phi}|\widehat{H}_{SOC}|\Phi\rangle\big|_{Q=0}\langle\bar{\Theta}_u|\Theta_v\rangle \\ &+ \sum_{Q_k}\left(\frac{\partial}{\partial Q_k}\langle\bar{\Phi}|\widehat{H}_{SOC}|\Phi\rangle\big|_{Q=0}\right)\langle\bar{\Theta}_u|Q_k|\Theta_v\rangle + \cdots\end{aligned} \tag{S11}$$

$$\langle\bar{\Phi}\bar{\Theta}_u|\widehat{H}_{BO}|\Phi\Theta_v\rangle = -\hbar^2\sum_k\left\langle\bar{\Phi}\bar{\Theta}_u\middle|\Phi\frac{\partial\Theta_v}{\partial\bar{Q}_k}\right\rangle = \sum_k\left\langle\bar{\Phi}\bar{\Theta}_u\middle|\Phi\left(\bar{\widehat{P}}_k\Theta_v\right)\right\rangle \tag{S12}$$

In the case of Ln^{3+} complexes, the strong coupling limit can be applied, since the ISC is a ligand-centered process. Also, the presence of the Ln^{3+} center induces relatively high SOCMEs at zero field, enabling the neglecting of Herzberg-Teller effects at room temperature ($k_BT \gg \hbar\omega_k$) resulting in the Franck-Condon type, Eq. S13, where only the

SOC term contributes significantly[28]. In this equation, the term $Z^{-1} = \left(\sum_{\nu=\{0_1,0_2,\cdots,0_N\}}^{\infty} e^{-\frac{E_\nu}{k_B T}}\right)^{-1}$ represents the inverse of the partition function.

$$W_{ST} = \frac{2\pi}{\hbar Z}\sum\sum_{k} e^{-\frac{E_\nu}{k_B T}} \left|\left\langle \bar{\Phi}\bar{\Theta}_u \middle| \Phi\left(\bar{\hat{P}}_k \Theta_\nu\right)\right\rangle\right|^2 \left|\langle \bar{\Phi}|\hat{H}_{SOC}|\Phi\rangle_{Q=0}\right|^2 |\langle \bar{\Theta}_u|\Theta_\nu\rangle|^2 \delta(E_\nu - \bar{E}_u) \quad \text{(S13a)}$$

$$W_{ST} = \frac{2\pi}{\hbar Z}|\langle \bar{\Phi}|\hat{H}_{SOC}|\Phi\rangle|^2 \sum_{u,\nu} e^{-\frac{E_\nu}{k_B T}} |\langle \bar{\Theta}_u|\Theta_\nu\rangle|^2 \delta(E_\nu - \bar{E}_u) \quad \text{(S13b)}$$

Under the framework of the harmonic oscillator model the vibrational wavefunction can be described as a product of individual vibrational modes considering each geometry coordinates (optimized singlet and triplet). The normal coordinates of the two states, i.e., initial and final states (S_1 and T_1, respectively, Q_k and $\overline{Q}_l$) are related to each other by an orthogonal Duschinsky rotation matrix ($J_{l,k}$) and a displacement vector (K_l) characterising the difference between the minimum and final state potential energy surface (Eqs. S14 – S15). Here, *L* denotes the eigenvector of the mass-weighted Hessian matrix. Within this approximation, the vibrational Hamiltonian is described by a displaced harmonic one-dimensional oscillator model (Eq. S16) where $\hat{P}_{j,k}^2$ and $\hat{Q}_{j,k}^2$ represents the *k-th* mass weighted nuclear normal momentum operator and normal coordinate of the *j-th* electronic state (*j* = S_1 and T_1). The generic potential energy surfaces of both S_1 and T_1 states is depicted in Figure S3, highlighting their displacement, which is essential in our approach to the calculation of the rates of ISC.

$$Q_l = \sum_k J_{l,k}\bar{Q}_k + K_l \quad \text{(S14)}$$

$$J_{l,k} = L_{i,l}^T \bar{L}_k;\ K_l = L_l^T\left(q_{0,l} - \bar{q}_{0,k}\right) \quad \text{(S15)}$$

$$\hat{H}_l = \frac{1}{2}\left(\hat{P}_l^2 + \omega_l^2 \hat{Q}_l^2\right);\ \bar{\hat{H}}_k = \frac{1}{2}\left(\bar{\hat{P}}_k^2 + \bar{\omega}_k^2 \bar{\hat{Q}}_k^2\right) \quad \text{(S16)}$$

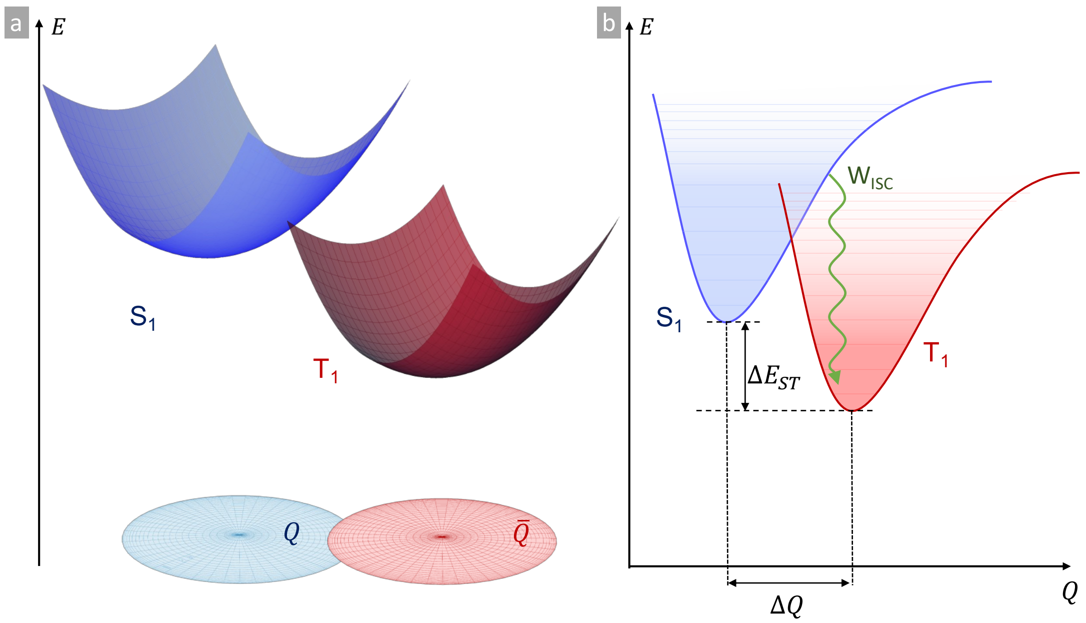

Figure S3. Generic potential energy surfaces (PES) for the S_1 and T_1 states in (a) three- and (b) two-dimensions. The relation between the normal coordinates of S_1 and T_1 is described by Eqs. S13 – S15. Q stands for the normal coordinates of the potential energy surface.

The computations of ISC rates under the time-independent perturbation theory (Eq. S13, strictly static regime) involves the calculation of the thermally averaged Franck-Condon (FC) integrals, yielding approaches demanding high computational power with elevated time-consuming. However, it is possible to avoid this computation by transforming the Fermi's golden rule into the Heisenberg picture, as proposed in previous studies.[26,27,30] In this regime, the Dirac δ function is Fourier transformed (Eq. S17) resulting in Eq. S18 when combined with Eq. S13. In this equation, $\omega_{if} = E_{if}/\hbar$, $\bar{\tau} = t$, $\tau = -t - \frac{i}{k_B T}$, and $\rho_{fi}^{(FC)}$ are the FC correlation functions represented by Eq. S19.

$$\delta(E_v - \bar{E}_u) = \frac{1}{2\pi} \int_{-\infty}^{+\infty} e^{i(E_v - \bar{E}_u)t/\hbar} dt \tag{S17}$$

$$W_{ST} = \frac{1}{Z\hbar^2} \left|\langle \bar{\Phi} | \hat{H}_{SOC} | \Phi \rangle\right|^2 \int_{-\infty}^{+\infty} e^{i\omega_{if}t} \rho_{ST}^{(FC)}(t) dt \tag{S18}$$

$$\rho_{ST}^{(FC)}(t) = Tr\left[e^{-i\bar{\tau}\bar{\hat{H}}} e^{-i\tau\hat{H}}\right] = \sum_{v,u} \langle \Theta_v | e^{-i\bar{\tau}\bar{E}_u} | \bar{\Theta}_u \rangle \left\langle \bar{\Theta}_u \middle| e^{-i\left(-t-\frac{i}{k_B T}\right)E_v} \middle| \Theta_v \right\rangle \tag{S19}$$

By considering the energy of the states as the eigenvalue of the vibrational Hamiltonian $\hat{H}$, the relation: $e^{-i\epsilon t}|\bar{\Theta}_u\rangle = e^{-i\hat{H}t}|\bar{\Theta}_u\rangle$ holds, imposing Eq. S20, which can be simplified using the resolution of identity: $\sum_u |\bar{\Theta}_u\rangle\langle\bar{\Theta}_u| = 1$, leading to the Eq. S21. This implies that the trace should be formulated in terms of the initial state (S_1).

$$\rho_{ST}^{(FC)}(t) = Tr\left[e^{-i\bar{\tau}\bar{\hat{H}}}e^{-i\tau\hat{H}}\right] = \sum_{v,u}\left\langle\Theta_v\left|e^{-it\bar{\hat{H}}}\right|\bar{\Theta}_u\right\rangle\left\langle\bar{\Theta}_u\left|e^{-i\left(-t-\frac{i}{k_BT}\right)\hat{H}}\right|\Theta_v\right\rangle \quad \text{(S20)}$$

$$\rho_{ST}^{(FC)}(t) = Tr\left[e^{-i\bar{\tau}\bar{\hat{H}}}e^{-i\tau\hat{H}}\right] = \sum_{v,u}\left\langle\Theta_v\left|e^{-i\bar{\tau}\bar{E}_u}e^{-i\left(-t-\frac{i}{k_BT}\right)E_v}\right|\Theta_v\right\rangle \quad \text{(S21)}$$

Under this picture, solving Eq. S21 becomes quite time-consuming, therefore transforming the traces to yield an analytical solution can become a simpler method to acquire the results. Therefore, following the approach by Shuai,[28] De Souza,[13] Pollak,[27] and Barone,[30] the trace in Eq. S21 can be solved in the continuous basis of the initial state normal coordinates Q at $t = t_0$, by including an extra set of coordinates Q' at time t and two sets of final coordinates $\bar{Q}$ and $\bar{Q}'$ with analogous definitions that leads to Eq S21. Here we have hidden the derivation steps for clarity which can be found in reference [13] for the fluorescence phenomenon ($S_n \rightarrow S_0$ photophysical process). All matrices defined in Eq. S22 are described in Eqs. S23a-d.

$$\rho_{ST}^{(FC)}(t) = \sqrt{\frac{det[\bar{a}a]}{det[B]det[B-AB^{-1}A]}}exp\left\{\frac{i}{\hbar}\left[D^TES(B-A)^{-1}GS^TD\right]\right\} \quad \text{(S22)}$$

$$A = \bar{a} + S^TaS \quad \text{(S231)}$$

$$B = \bar{b} + S^TbS \quad \text{(S23b)}$$

$$G = \bar{b} - \bar{a} \quad \text{(S23c)}$$

$$E = b - a \quad \text{(S23d)}$$

In this set of equations a and b are diagonal matrices with elements $a_k = \omega_k/sin(\hbar\omega_k\tau)$, and $b_k = \omega_k/tan(\hbar\omega_k\tau)$. Under this circumstance the exponential part of Eq. S22 can be simplified by grouping all matrices into a new set defined in Eq. S24, yielding the reduced expression of the Franck-Concon density of states using the time-dependent approach (Eq. S25).

$$K = \begin{bmatrix} B & -A \\ -A & B \end{bmatrix}_{2\times 2N}; \ \underline{F} = \left[\underline{D}^TES\underline{D}^TES\right]^T_{1\times 2N} \quad \text{(S24)}$$

$$\rho_{ST}^{(FC)}(t) = \sqrt{\frac{det[\bar{a}a]}{det[K]}}exp\left\{\frac{i}{\hbar}\left[-\frac{1}{2}\underline{F}^TK^{-1}\underline{F} + \underline{D}^TE\underline{D}\right]\right\} \quad \text{(S25)}$$

The final rates in this approach are computed by a Fourier transform of the correlation function on a time grid by choosing the time interval and maximum time in which the correlation function will be calculated.

Local vibrational analysis

The normal vibrational modes are delocalized across the entire molecule,[32] imposing difficulties in accurately analyzing the contributor fragments to its formation, which can be solved by local vibrational mode (LMV) theory,[22] whose foundations were laid by Konkoli and Cremer.[33] The cornerstone of the theory lies in harnessing local vibrational modes including intrinsic bond properties from normal vibrational modes.[22] Besides, the theory also furnishes a unique method to analyze vibrational spectra by decomposing a specific normal vibrational mode into local mode components, known as characterization of normal modes (CNM) procedure.[24,34] This procedure is possible due to an essential one-to-one relationship between the normal mode counterparts and the complete set of non-redundant local modes via an adiabatic connection scheme (ACS).[35] The subtleties of LMV theory have been extensively described in recent reviews,[21,22] and although several physical and mathematical basis are needed to accurately describe this theory, in this section we aim to highlight the main aspects of the method. The local mode vectors a_n associated with the internal coordinates q_n is determined by using the normal vibrational mode vectors in internal coordinates (d_n) and the diagonal force constant matrix (K), as summarized in Eq. S26, where the local mode force constant is determined based on the local mode vector (Eq. S27).

$$a_n = \frac{K^{-1}d_n^{\dagger}}{d_n K^{-1} d_n^{\dagger}} \tag{S26}$$

$$k_n^a = a_n^{\dagger} K a_n = \left(d_n K^{-1} d_n^{\dagger}\right)^{-1} \tag{S27}$$

Within this framework all normal vibrational modes can be decomposed into local vibrational mode percentage contribution whenever associated with absorption peaks, from a non-redundant set of local vibrational modes using the CNM protocol. The basis of this procedure lies in the overlapping of modes encoding by $S_{n\mu}$ (Eq. S28), where a_n^x represents the local vibrational mode, F^x the Hessian matrix and $I_\mu$the normal vibrational mode in Cartesian coordinates. In this sense, the contribution (weighted percentage local mode contribution) of a_n^x to the normal mode I_μ is determined by Eq. S29.

$$S_{n\mu} = \frac{\left\langle a_n^x \middle| F^x \middle| I_\mu \right\rangle^2}{\left\langle a_n^x \middle| F^x \middle| a_n^x \right\rangle \left\langle I_\mu \middle| F^x \middle| I_\mu \right\rangle} \tag{S28}$$

$$C_{n\mu}^{\%} = \frac{S_{n\mu}}{\sum_m^{N_{vib}} S_{m\mu}} 100 \tag{S29}$$

Supplementary note S3 – Structural analysis

The geometry optimization of the complexes at the S_1 and T_1 levels yielded distortions when comparing both structures, which can be found in **Figure 1** of the main text, where the degree of distortion was qualitatively assessed by the root mean square deviation (RMSD) described in Eq. S29, calculated by neglecting translations between the center of mass of the molecule to reduce the possibility of incorrect values. In Eq. S30, v_i is the atomic coordinate vector related to the T_1 geometry taking the europium as reference, while w_i is the atomic coordinate vector related to the S_1 geometry. The obtained values are summarized in **Table S1**. These distortions promoted variations in the bond length within the structures, described in **Tables S2 – S5**.

$$RMSD = \sqrt{\frac{1}{n}\sum_{i=1}^{n}\|v_i - w_i\|^2}$$
$$= \sqrt{\frac{1}{n}\sum_{i=1}^{n}\left((v_{ix} - w_{ix})^2 + \left(v_{iy} - w_{iy}\right)^2 + (v_{iz} - w_{iz})^2\right)} \quad \text{(S30)}$$

Table S1. Values of RMSD calculated according to equation S30 for each complex object of this study.

Complex	Value of RMSD (Å)
**[Eu(tta)$_3$(H$_2$O)$_2$]**	0.155
**[Eu(tta)$_4$]$^-$**	0.356
**[Eu(NO$_3$)$_3$(phen)$_2$]**	0.234
**[Eu(PyRCF$_3$)$_3$(phen)]**	0.501

Table S2. Values of bond length for the atoms coordinated to Eu^{3+} in both S_1 and T_1 geometries for the [Eu(tta)$_3$(H$_2$O)$_2$] complex. The O(1) and O(2) are assigned to the coordinated water molecules while the other oxygens are related to the *tta* ligand.

**[Eu(tta)$_3$(H$_2$O)$_2$**	**Bond length / Å**	
	**S_1**	**T_1**
**Eu−O(1)**	2.44	2.42
**Eu−O(2)**	2.18	2.14
**Eu−O(3)**	2.77	2.70
**Eu−O(4)**	2.13	2.13
**Eu−O(5)**	2.10	2.09
**Eu−O(6)**	2.15	2.13
**Eu−O(7)**	2.04	2.02
**Eu−O(8)**	2.16	2.13

Table S3. Values of bond length for the atoms coordinated to Eu^{3+} in both S_1 and T_1 geometries for the [Eu(tta)$_4$]$^-$ complex.

**[Eu(tta)$_4$]$^-$**	**Bond length / Å**	
	**S_1**	**T_1**
**Eu−O(1)**	2.75	2.53
**Eu−O(2)**	2.41	2.40
**Eu−O(3)**	2.36	2.25
**Eu−O(4)**	2.39	2.35
**Eu−O(5)**	2.39	2.39
**Eu−O(6)**	2.43	2.23
**Eu−O(7)**	2.60	2.37
**Eu−O(8)**	2.35	2.25

Table S4. Values of bond length for the atoms coordinated to Eu^{3+} in both S_1 and T_1 geometries for the $[Eu(NO_3)_3(phen)_2]$ complex. The N(1) and N(2) relates to the one phen ligand while N(3) and N(4) is related to another phen ligand.

**$[Eu(NO_3)_3(phen)_2]$**	**Bond length / Å**	
	**S_1**	**T_1**
**Eu−O(1)**	2.49	2.45
**Eu−O(2)**	2.54	2.50
**Eu−O(3)**	2.48	2.44
**Eu−O(4)**	2.54	2.49
**Eu−O(5)**	2.53	2.49
**Eu−O(6)**	2.55	2.50
**Eu−N(1)**	2.61	2.53
**Eu−N(2)**	2.70	2.64
**Eu−N(3)**	2.63	2.59
**Eu−N(4)**	2.69	2.65

Table S5. Values of bond length for the atoms coordinated to Eu^{3+} in both S_1 and T_1 geometries for the $[Eu(PyRCF_3)_3(phen)]$ complex.

**$[Eu(tta)_4]^-$**	**Bond length / Å**	
	**S_1**	**T_1**
**Eu−O(1)**	2.37	2.35
**Eu−O(2)**	2.37	2.31
**Eu−O(3)**	2.39	2.36
**Eu−O(4)**	2.36	2.35
**Eu−O(5)**	2.50	2.34
**Eu−O(6)**	2.72	2.40
**Eu−N(1)**	2.64	2.51
**Eu−N(2)**	2.63	2.51

Furthermore, the bond angles exhibited modifications when going from the S_1 to the T_1 geometries for all complexes, as shown in **Figure S4**.

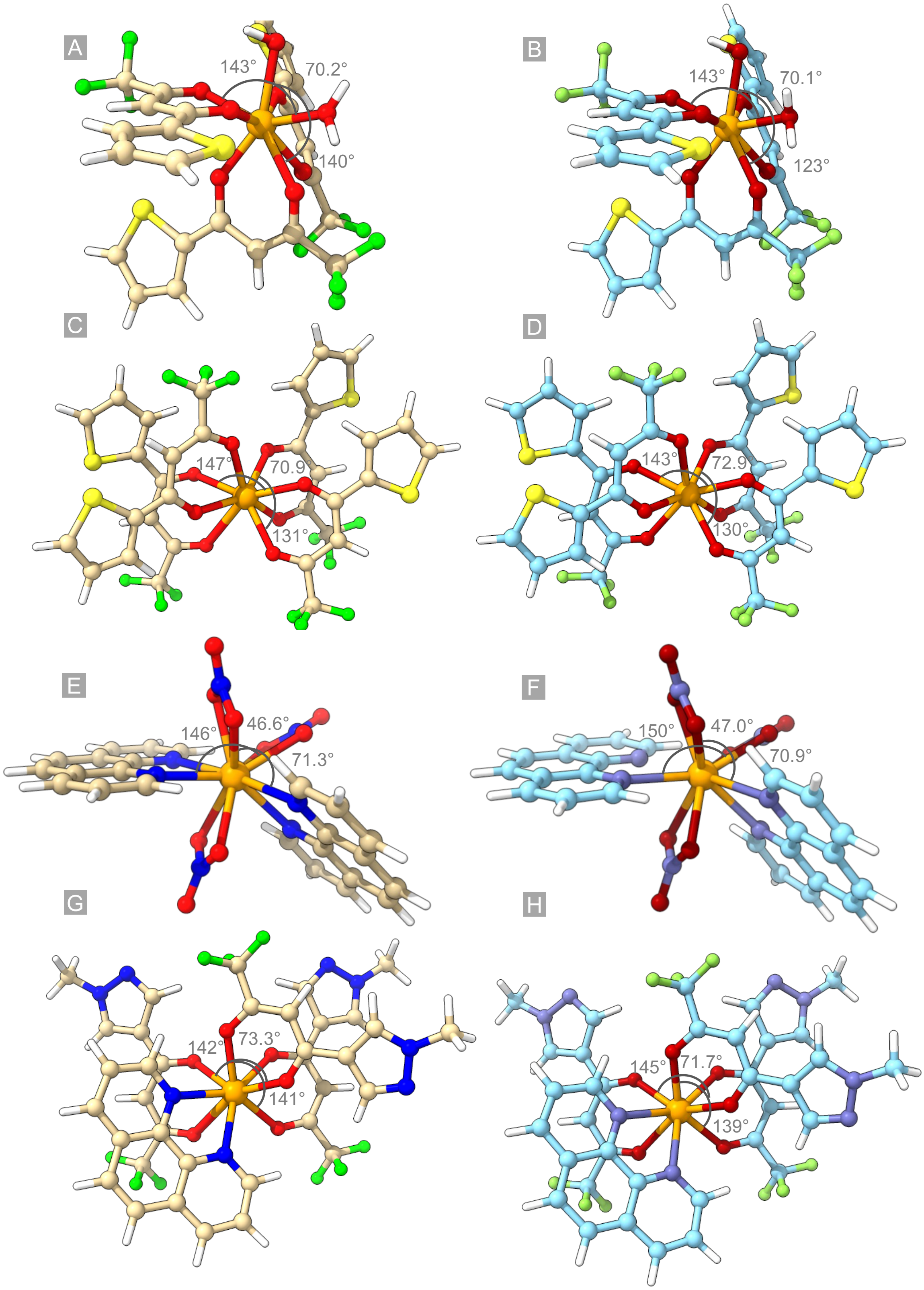

Figure S4. Bond angle distortions in the atoms coordinated to Eu^{3+} for $[Eu(tta)_3(H_2O)_2]$ at the (a) S_1 and (b) T_1; $[Eu(tta)_4]^-$ at the (C) S_1 and (d) T_1 geometries; $[Eu(NO_3)_3(phen)_2]$ at the (e) S_1 and (f) T_1 geometries, and $[Eu(PyrCF_3)_3(phen)]$ at the (g) S_1 and (h) T_1 geometries. All structures from a–h possess a C_1 symmetry point group. S_1 geometry: blue = nitrogen, light yellow = carbon, lime green = fluorine, orange = europium, red = oxygen, yellow = sulfur, white = hydrogen. T_1 geometry: pale blue = nitrogen, light blue = carbon, green = fluorine, orange = europium, dark red = oxygen, dark yellow = sulfur, white = hydrogen.

Supplementary Note S4 – Electronic structure

The molecular orbitals of the studied compounds are pivotal in understanding their photophysical behavior. Due to the non-negligible spin-orbit coupling of Ln^{3+}-based compounds, S_1 and T_1 states are not pure, and they must be generated by a combination of several molecular orbitals. In light of this guidance, this supplementary note aims to provide the fundamental of these states as well as their impact on the rates of intersystem crossing. The molecular orbitals (MO) involved in S_1 and T_1 states are represented in Fig S5 – Fig S8, where the light blue and light wine backgrounds are analogue to the background of Fig S2 representing the singlet and triplet states, respectively.

Table S6. Percentage contribution of the molecular orbitals to the formation of S_1 and T_1 states. Only the MOs with contribution higher than 1%. L = LUMO.

Complex	S_1 state	T_1 state
**[Eu(tta)$_3$(H$_2$O)$_2$]**	59% L; 29%L+2	27% L; 36% L+1; 22% L+2
**[Eu(tta)$_4$]$^-$**	49% L; 2% L+1; 34% L+3;	11% L; 65% L+; 21% L+2
**[Eu(NO$_3$)$_3$(phen)$_2$]**	69% L; 20% L+1; 6% L+2	02% L; 66% L+1; 18% L+3
**[Eu(PyrCF$_3$)$_3$(phen)]**	2% L; 82% L+1; 10% L+4	81% L+1; 12% L+2

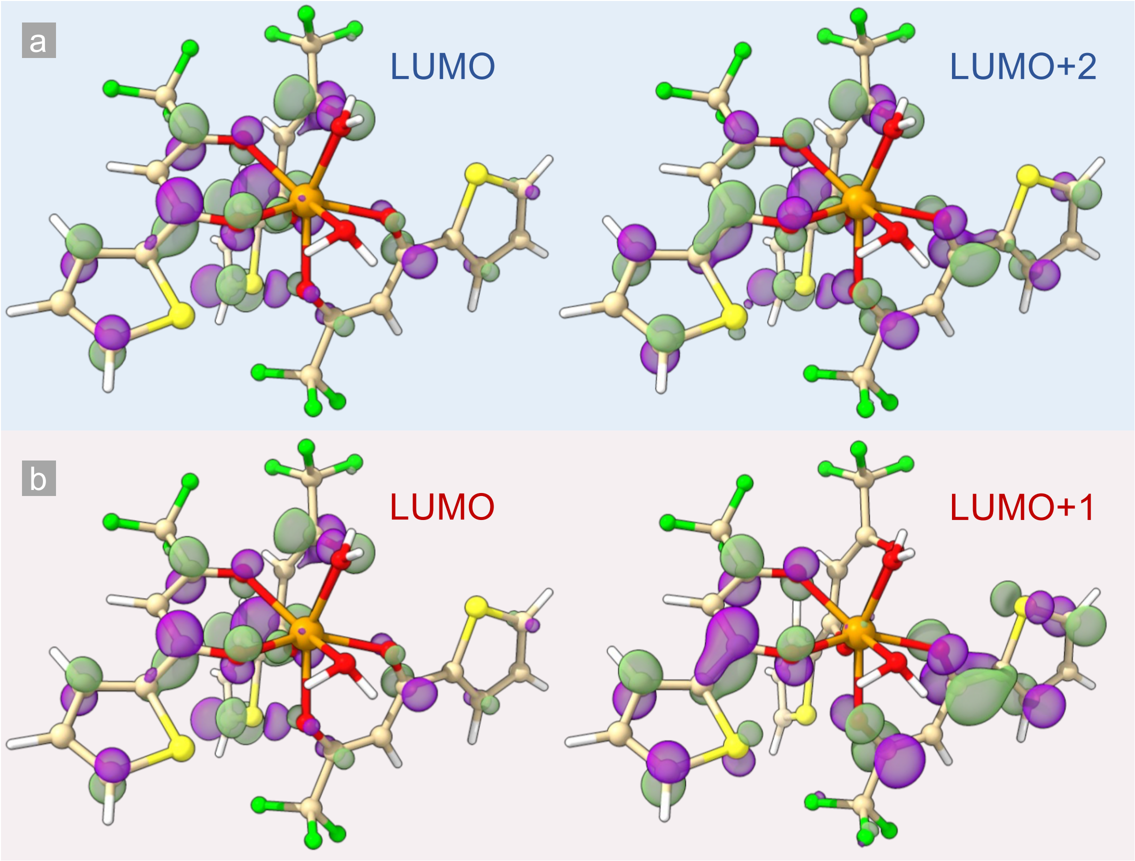

Figure S5. Most contributing molecular orbitals to the (a) S_1 and (b) T_1 states for the [Eu(tta)$_3$(H$_2$O)$_2$] complex. Light yellow = carbon, lime = fluorine, orange = europium, red = oxygen, white = hydrogen.

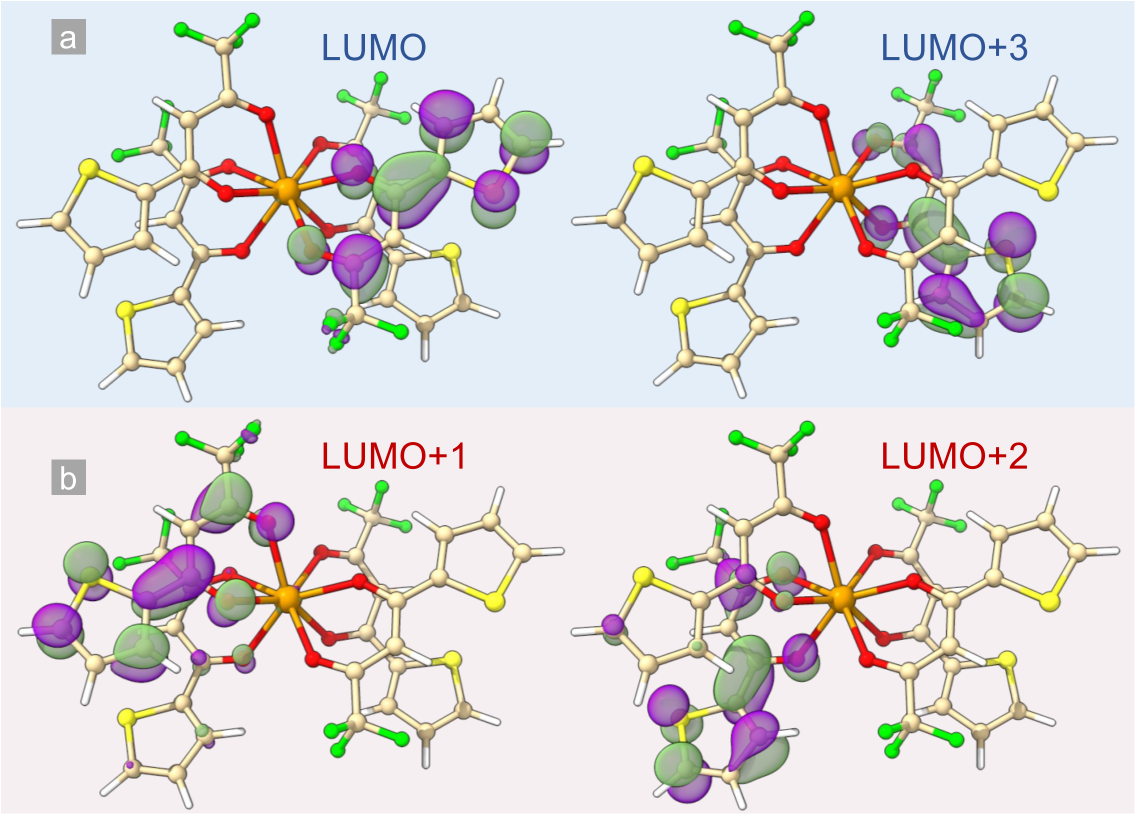

Figure S6. Most contributing molecular orbitals to the (a) S_1 and (b) T_1 states for the $[Eu(tta)_4]^-$ complex. Light yellow = carbon, lime = fluorine, orange = europium, red = oxygen, white = hydrogen.

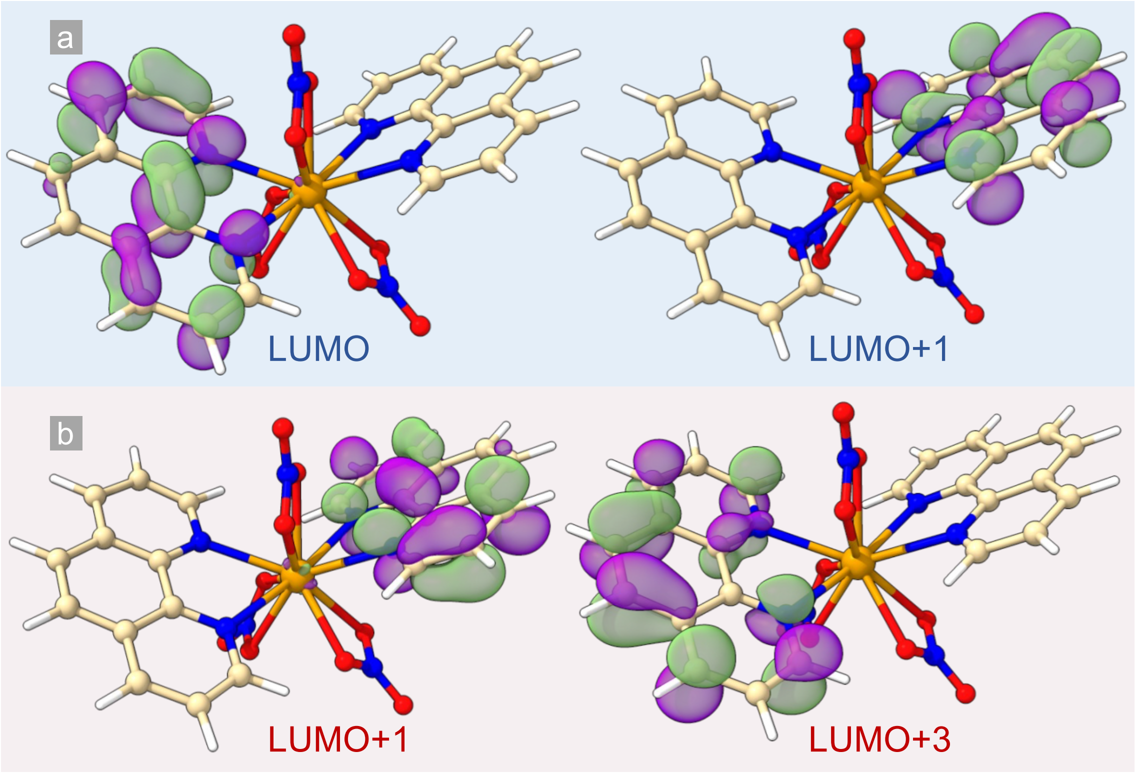

Figure S7. Most contributing molecular orbitals to the (a) S_1 and (b) T_1 state for the $[Eu(NO_3)_3(phen)_2]$ complex. Blue = nitrogen, light yellow = carbon, orange = europium, red = oxygen, white = hydrogen.

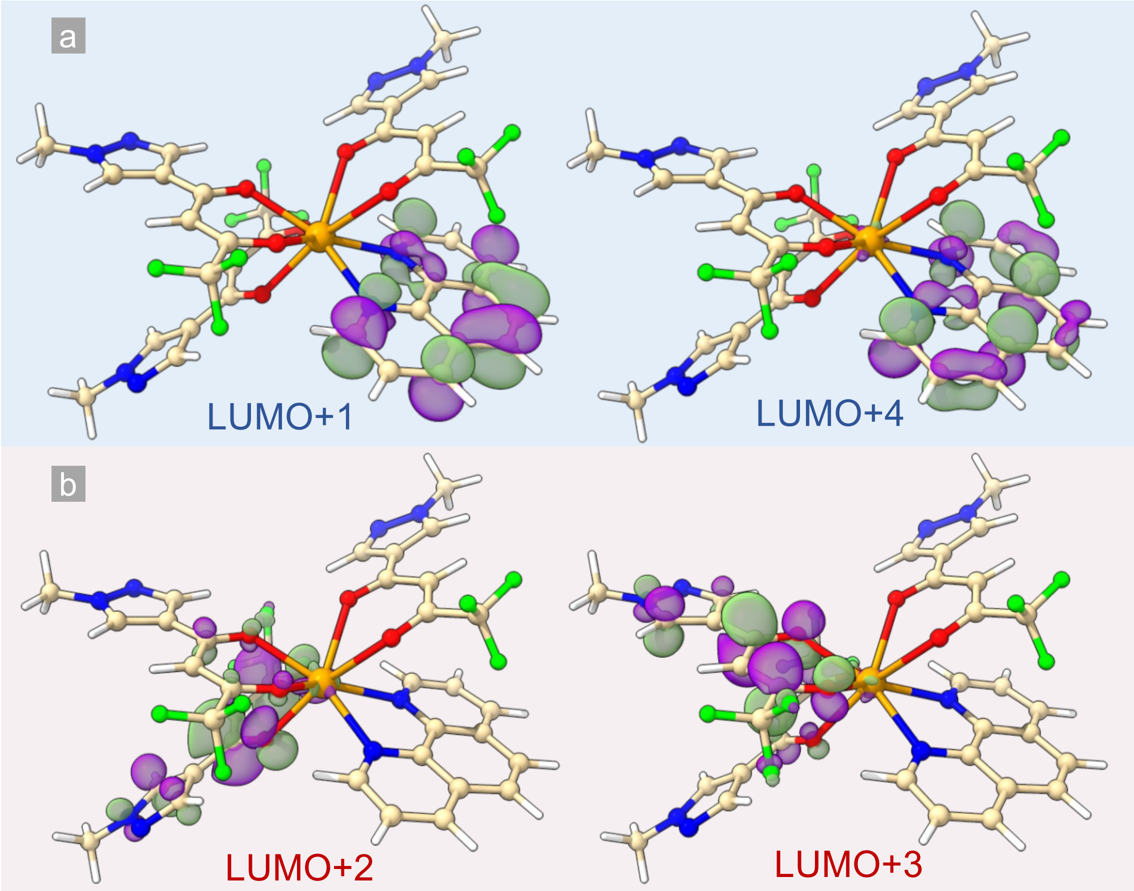

Figure S8. Most contributing molecular orbitals to the (a) S_1 and (b) T_1 states for the $[Eu(PyrCF_3)_3(phen)]$ complex. Blue = nitrogen, light yellow = carbon, lime = fluorine, orange = europium, red = oxygen, white = hydrogen.

Table S7. Excited state character of ligand centered S_1 and T_1 states based on the molecular orbitals depicted in **Figures S5 – S8**. All characters were assigned by inspecting the isosurfaces in Figures S5 – S8.

Complex	Excited state character	
	S_1	T_1
$[Eu(tta)_3(H_2O)_2]$	$^1n\pi^*$ / $^1n\pi^*$	$^3n\pi^*$ / $^3\pi\pi^*$
$[Eu(tta)_4]^-$	$^1n\pi^*$ / $^1n\pi^*$	$^3\pi\pi^*$ / $^3\pi\pi^*$
$[Eu(NO_3)_3(phen)_2]$	$^1n\pi^*$ / $^1\pi\pi^*$	$^3\pi\pi^*$ / $^3n\pi^*$
$[Eu(PyrCF_3)_3(phen)]$	$^1n\pi^*$ / $^1n\pi^*$	$^3\pi\pi^*$ / $^3\pi\pi^*$

Supplementary Note S5 - El-Sayed rules for intersystem crossing and electronic structure of lanthanides

Analysing the rates of intersystem crossing from a quantitative perspective can very often be quite untrivial for coordination compounds owing to the large complexity of excited-state dynamics. Because of such phenomena, qualitative analysis is a simpler way to comprehend their photophysical features, and this is where El-Sayed´s rules come into play. From Eq. S16, we observe that the ISC rates are directly proportional to the spin-orbit coupling matrix elements (SOCMEs), and from a pure electronic point of view, i.e., neglecting vibrational considerations, the El-Sayed rules can be used to qualitatively predict the order of SOCMEs.

In his work, El-Sayed described that to harness effective SOCME, any change in spin ($S_1 \rightarrow T_1$) must be accompanied by a corresponding change in angular momentum to conserve the angular momentum. This rule is represented in Figure S9a, while the change in angular momentum due to its projection across an axis is depicted in Figure S9b.

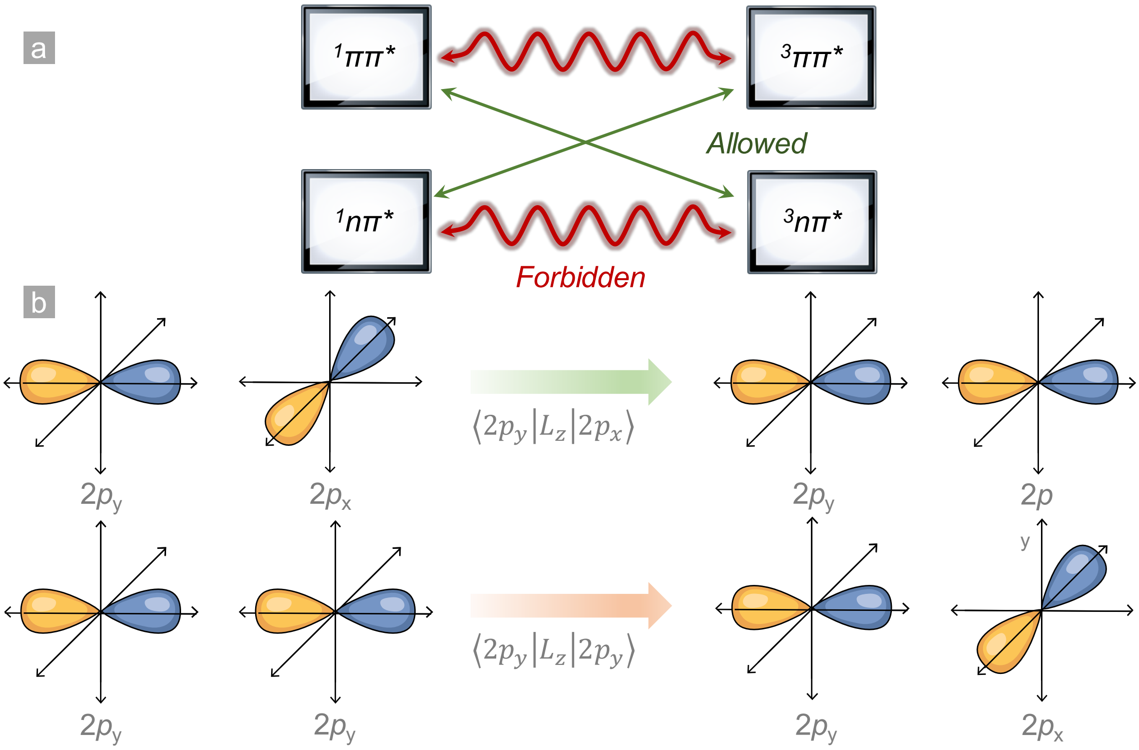

Figure S9. General case of El-Sayed rules, where transitions between molecular orbitals of same character are forbidden (a) and rotation property of the orbital angular momentum (b).

The electronic structure of lanthanide ions must be highlighted in this section, as the spin-orbit interaction in these ions have almost the same magnitude of the electrostatic interaction (Figure S10), turning S and L not no longer pure quantum numbers, relaxing the El-Sayed rules. For objective purposes only the spin-orbit coupling term in the energy diagram of lanthanides will be highlighted in this supplementary note.

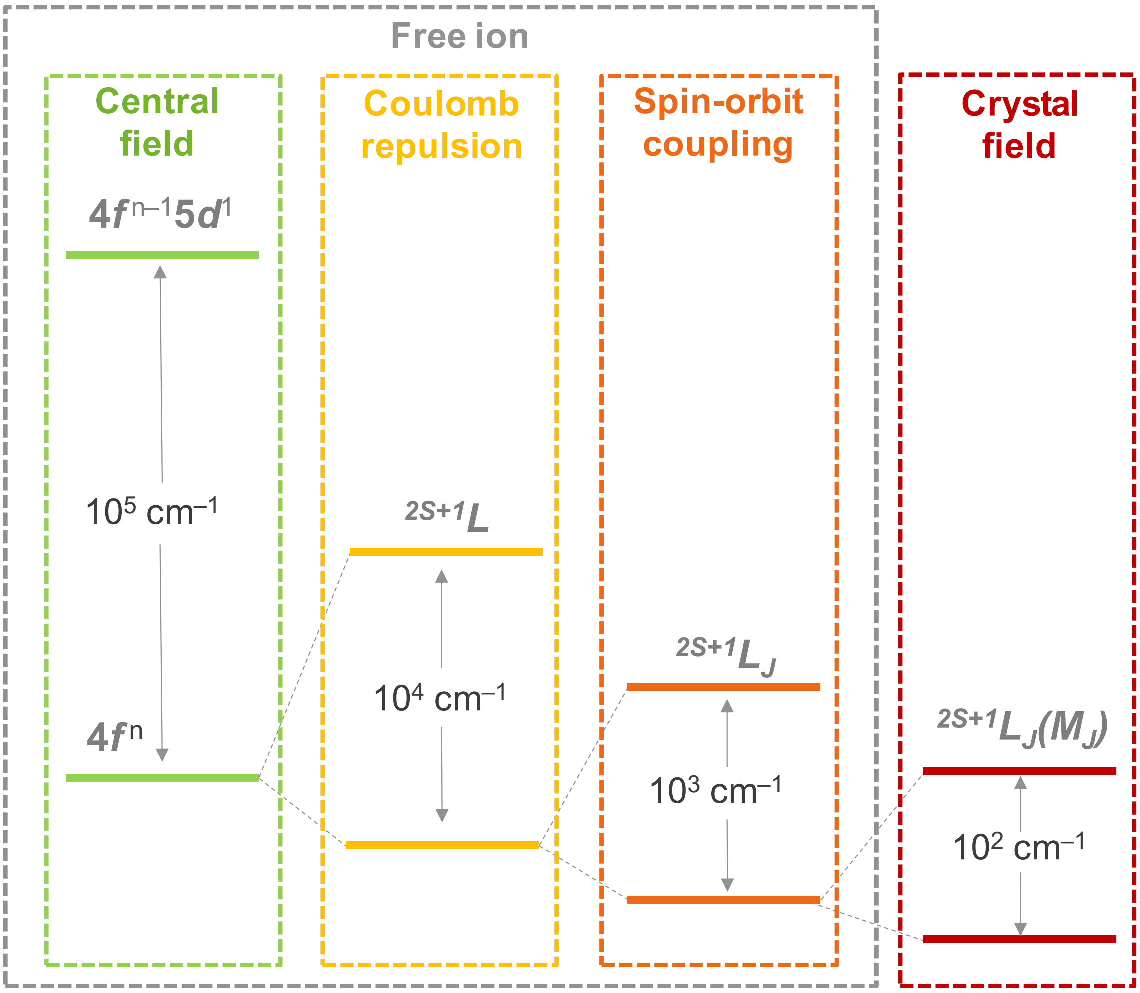

Figure S10. Contributions of different factors on the energy levels of trivalent lanthanide ions that experiences a specific coordination environment. The order of magnitude of each interaction is shown in wavenumbers varying from 10^5 – 10^2 due to the interactions highlighted in each color. *S*, *L*, and *J* are the quantum numbers describing the spin angular momentum, orbital angular momentum, and total angular momentum, respectively. $^{2S+1}L_J$ is the representation in the Russel-Saunders scheme for the term symbol while M_J is the azimuthal projection of *J*. Adapted from reference [36].

The Hamiltonian describing the interactions occurring in Figure S10 can be partitioned into free-ion and crystal field ($\hat{H}_{FI}$ and $\hat{H}_{CF}$, respectively), where the free-ion considers only the electrostatic and spin-orbit interactions ($\hat{H}_{FI} = \hat{H}_{ee} + \hat{H}_{SO}$). Focusing on the last, the spin-orbit interactions is depicted in Eq. S31, where r_i is the radial coordinate of the *i*-th electron, $\hbar$ the reduced planck's constant, m the mass of the electron, c the speed of light, $U(r_i)$ the central field potential, s_i and l_i represents the spin and orbital angular momentum, respectively.

$$\hat{H}_{SO} = \sum_{i=1}^{N} \xi \left(\frac{\hbar^2}{m^2 c^2 r_i}\right) \left\{\frac{dU(r_i)}{dr_i}\right\} (s_i \cdot l_i) \qquad \text{(S31)}$$

The spin-orbit interaction yields off-diagonal elements to the Hamiltonian in the eigenvalue problem considering the intermediate coupling scheme, as it is not diagonal in the basis of *L* and *S*. Therefore, describing the matrix elements of $\hat{H}_{SO}$ is crucial, since

this effect mix states with the same *J*, leading us to Eq. S32, where Eq. S33 describes the reduced matrix elements of the spin-orbit interaction.

$$\langle 4f^N SLJ | \hat{H}_{SO} | 4f^N S'L'J \rangle = \zeta(-1)^{J+L+S'} 2\sqrt{21} \times \begin{Bmatrix} S & S' & 1 \\ L' & L & J \end{Bmatrix} \langle l^N SL || V^{(11)} || 3^N S'L' \rangle \tag{S32}$$

$$\langle 3^N SL || V^{(11)} || 3^N S'L' \rangle = N\sqrt{1/2(2S+1)(2L+1)(2S'+1)(2L'+1)} \times \sum_{\psi(3^{N-1})} (3^{N-1}\bar{S}\bar{L} | 3^N SL) \begin{Bmatrix} S & S' & 1 \\ 1/2 & 1/2 & \bar{S} \end{Bmatrix} \begin{Bmatrix} L & L' & 1 \\ 3 & 3 & \bar{L} \end{Bmatrix} (-1)^{\bar{S}+\bar{L}+S+L+11/2} \tag{S33}$$

In this sense, non-vanishing matrix elements are observed even for transitions forbidden by the El-Sayed rules, leading to values in the same order as the transitions allowed by the same rules.

Supplementary Note S6 – Vibronic properties

As discussed in the main text, the vibrational density of states is crucial in understanding the rates of intersystem crossing as well as the vibronic coupling. Thus, we analyze this phenomenon by two main properties, the Huang-Rhys factor and Reorganization energies. Table S8 – S11 summarizes the obtained values for the series of complex under study.

Table S8. Huang-Rhys (HR) factor and reorganization energies for [Eu(tta)$_3$(H_2O)$_2$] per normal vibrational mode.

Normal mode index	Huang-Rhys factor	Reorganization energy / cm^{-1}
1	0	0
2	0.000232	0.0056
3	0.002452	0.08284
4	0.001016	0.04116
5	0.000712	0.03124
6	0.000108	0.00516
7	0.000268	0.01344
8	0	0.00004
9	0.000032	0.00188
10	0.00036	0.02328
11	0.000008	0.00052
12	0.000284	0.02164
13	0.000096	0.00788
14	0.000228	0.02028
15	0.000004	0.00056
16	0.000004	0.00044
17	0.000068	0.00732
18	0.000212	0.02572
19	0.000724	0.08892
20	0.000036	0.00476
21	0.000404	0.06096
22	0.000056	0.00876
23	0.000156	0.02508
24	0.000224	0.03856
25	0.000192	0.03452
26	0.000192	0.03788
27	0.0001	0.02024
28	0.000068	0.0144
29	0.000144	0.03124
30	0.00002	0.00456
31	0.000304	0.06804
32	0.000264	0.0598
33	0	0
34	0.00028	0.06868
35	0.000692	0.17812
36	0.000496	0.13164
37	0.001016	0.2704
38	0.000708	0.19632

**39**	0.000092	0.02716
**40**	0.000004	0.0018
**41**	0.000056	0.01688
**42**	0.000568	0.18524
**43**	0.000016	0.00524
**44**	0.0008	0.27408
**45**	0.000252	0.08788
**46**	0.002408	0.84504
**47**	0.000008	0.0024
**48**	0.000252	0.09352
**49**	0.002652	1.02104
**50**	0	0.0002
**51**	0.011184	5.04804
**52**	0.000704	0.33152
**53**	0.000028	0.01332
**54**	0.000076	0.0362
**55**	0.073932	35.94652
**56**	0.134252	66.67564
**57**	0.006128	3.09412
**58**	0.001532	0.78788
**59**	0.01914	9.9468
**60**	0.000876	0.4594
**61**	0.001124	0.59996
**62**	0.001156	0.63288
**63**	0.000008	0.00464
**64**	0.000212	0.12172
**65**	0.002252	1.30764
**66**	0.001992	1.1608
**67**	0.0001	0.0594
**68**	0.00038	0.22528
**69**	0.006552	3.90628
**70**	0.0005	0.31432
**71**	0.00144	0.91224
**72**	0.000348	0.22376
**73**	0.011976	7.69012
**74**	0.011712	7.61
**75**	0.000052	0.03484
**76**	0.005808	3.82124
**77**	0.023336	15.82164
**78**	0.000144	0.10088
**79**	0.000428	0.29708
**80**	0.004936	3.43492
**81**	0.000948	0.66684
**82**	0.007252	5.13164
**83**	0.000296	0.21168
**84**	0.000012	0.00772
**85**	0.000636	0.47428
**86**	0.000012	0.00788

**87**	0.000072	0.05564
**88**	0.00048	0.36536
**89**	0	0.00148
**90**	0	0.00024
**91**	0.000064	0.05012
**92**	0.001712	1.33972
**93**	0.000356	0.28144
**94**	0.000016	0.01192
**95**	0.000052	0.04304
**96**	0.000048	0.04052
**97**	0.000236	0.19948
**98**	0.00002	0.01568
**99**	0.000008	0.00592
**100**	0.000136	0.12016
**101**	0.000712	0.63212
**102**	0	0.00068
**103**	0.000544	0.50384
**104**	0	0.00096
**105**	0.000036	0.03308
**106**	0	0.00068
**107**	0.000696	0.69276
**108**	0.000936	0.95336
**109**	0.000004	0.00516
**110**	0.000108	0.11424
**111**	0.000264	0.28548
**112**	0.000016	0.01676
**113**	0	0.00016
**114**	0.000016	0.01908
**115**	0.000044	0.04928
**116**	0.000692	0.76048
**117**	0.000376	0.41748
**118**	0.003088	3.56812
**119**	0.000004	0.00604
**120**	0.000968	1.13204
**121**	0.000348	0.40968
**122**	0.000088	0.1038
**123**	0.000136	0.163
**124**	0.000276	0.33604
**125**	0	0.00192
**126**	0.000008	0.0086
**127**	0.000056	0.06948
**128**	0	0.00016
**129**	0.000044	0.05676
**130**	0.000044	0.0584
**131**	0	0
**132**	0.00002	0.02496
**133**	0.000876	1.18852
**134**	0	0.00004

**135**	0.00002	0.02648
**136**	0.000076	0.10612
**137**	0.000008	0.00988
**138**	0	0.00212
**139**	0.002068	2.98404
**140**	0.010556	15.47152
**141**	0.000504	0.74128
**142**	0.00644	9.55564
**143**	0.067972	101.56304
**144**	0.22212	333.94432
**145**	0.009212	13.9128
**146**	0.003192	4.93088
**147**	0.000232	0.356
**148**	0.009132	14.3528
**149**	0.001924	3.05096
**150**	0.003016	4.82832
**151**	0.031704	50.84328
**152**	0.004252	6.92272
**153**	0.000224	0.38556
**154**	0	0.00012
**155**	0	0
**156**	0	0.00068
**157**	0	0
**158**	0	0
**159**	0	0.00024
**160**	0	0.0006
**161**	0	0
**162**	0	0.00164
**163**	0.000004	0.01736
**164**	0	0.0022
**165**	0	0.00008
**166**	0.559108	2041.3572
**167**	0.008652	32.08816
**168**	0.040976	157.49896
**169**	0.232456	917.4492

Table S9. Huang-Rhys (HR) factor and reorganization energies for $[Eu(tta)_4]^-$ per normal vibrational mode.

Normal mode index	Huang-Rhys factor	Reorganization energy / cm^{-1}
**1**	0.000005	0.000102
**2**	1.667E-06	3.13E-05
**3**	0.000035	0.000883
**4**	0.0011892	0.035851
**5**	9.167E-05	0.003025
**6**	0.0008267	0.028643
**7**	1.667E-06	7.82E-05
**8**	0.00017	0.007255
**9**	0.0031325	0.140252
**10**	0.000135	0.006332
**11**	8.417E-05	0.004206
**12**	0.0001125	0.006309
**13**	0.0000925	0.00523
**14**	0.0009183	0.05387
**15**	8.167E-05	0.004987
**16**	0.0001625	0.011054
**17**	2.167E-05	0.001493
**18**	8.333E-07	7.82E-05
**19**	9.333E-05	0.007208
**20**	9.083E-05	0.007122
**21**	0.0000525	0.004268
**22**	0.0000025	0.000203
**23**	0.0001092	0.010694
**24**	0.0019842	0.195865
**25**	9.917E-05	0.010077
**26**	4.333E-05	0.004862
**27**	0.00083	0.094731
**28**	0.0013125	0.1543
**29**	0.0000025	0.000289
**30**	0.00062	0.082231
**31**	0.0024508	0.330574
**32**	0.0031333	0.480597
**33**	0.0009	0.138579
**34**	0.0079858	1.330605
**35**	0.0027842	0.510124
**36**	0.0023875	0.440807
**37**	0.0099692	1.872811
**38**	0.0087792	1.666284
**39**	0.0153867	2.939728
**40**	0.0008908	0.177205
**41**	0.0024608	0.50523
**42**	0.0001825	0.038164
**43**	0.0066717	1.426954
**44**	0.0003175	0.068918
**45**	0.0313733	7.1649

**46**	0.0086808	2.003401
**47**	0.0021467	0.508826
**48**	0.0001417	0.033951
**49**	0.0001767	0.043551
**50**	0.0024633	0.636492
**51**	0.0010508	0.293644
**52**	0.0001633	0.046576
**53**	0.00167	0.485014
**54**	0.0558833	16.60532
**55**	0.0081542	2.502556
**56**	0.00195	0.606762
**57**	0.0047275	1.507356
**58**	0.1233883	40.32364
**59**	0.0226858	7.585475
**60**	0.020465	6.902259
**61**	0.0068142	2.30978
**62**	0.001345	0.558701
**63**	0.0118233	4.95498
**64**	0.0031792	1.361867
**65**	0.0000275	0.011671
**66**	0.0016658	0.721607
**67**	0.0936483	40.80968
**68**	0.0002275	0.099765
**69**	0.0047708	2.10326
**70**	0.0093117	4.220052
**71**	0.0003033	0.138508
**72**	0.0458333	21.59039
**73**	5.167E-05	0.024984
**74**	0.0008042	0.38879
**75**	0	1.56E-05
**76**	0.0000025	0.001048
**77**	2.833E-05	0.013962
**78**	0.0006667	0.346607
**79**	0.0060908	3.203018
**80**	0.055125	29.86302
**81**	0.0096292	5.224656
**82**	0.001735	0.948014
**83**	0.0055367	3.0255
**84**	0.000205	0.112047
**85**	0.0060417	3.319708
**86**	0.0003492	0.194575
**87**	0.003575	2.003252
**88**	0.001415	0.803338
**89**	3.833E-05	0.022029
**90**	0.0676158	39.03655
**91**	0.1302542	75.35899
**92**	0.0008533	0.536703
**93**	0.0174275	11.27616

**94**	0.0303158	19.66161
**95**	0.0001217	0.080316
**96**	0.0383008	25.30171
**97**	2.917E-05	0.019442
**98**	0.0016225	1.077775
**99**	0.0012308	0.819942
**100**	0.0013283	0.895536
**101**	0.0002617	0.180019
**102**	0.0001192	0.082208
**103**	0.0292458	20.2813
**104**	0.0032642	2.269903
**105**	0.0001658	0.117487
**106**	0.0020542	1.458927
**107**	0.0001408	0.100915
**108**	0.0021217	1.518965
**109**	0.005615	4.047483
**110**	0.0035233	2.5779
**111**	0.0016675	1.237031
**112**	0.0061525	4.566096
**113**	0.225975	167.8906
**114**	0.0721483	53.78712
**115**	0.010725	8.186961
**116**	0.0021842	1.670912
**117**	0.0085058	6.531129
**118**	0.06054	47.59156
**119**	0.0033475	2.644841
**120**	0.00004	0.0327
**121**	0.0001842	0.149093
**122**	0.0033208	2.726642
**123**	0.0044692	3.679346
**124**	0.0001458	0.121459
**125**	0.00135	1.127423
**126**	0.0064792	5.538891
**127**	0.1597008	139.1728
**128**	2.083E-05	0.018355
**129**	0.0044033	3.914063
**130**	0.0003892	0.34667
**131**	0.0345933	30.90763
**132**	0.0014533	1.318652
**133**	1.417E-05	0.012531
**134**	0.04061	39.28316
**135**	0.14391	140.8978
**136**	5.667E-05	0.055863
**137**	0.0002608	0.257356
**138**	0.07812	78.057
**139**	0.0090492	9.089063
**140**	0.1092358	110.4038
**141**	0.0003783	0.382888

**142**	0.0010692	1.085006
**143**	0.01919	19.57386
**144**	0.0017283	1.782004
**145**	0.0337958	34.98687
**146**	0.0049917	5.241432
**147**	0.0096583	10.17988
**148**	2.083E-05	0.022452
**149**	8.833E-05	0.096482
**150**	0.0003175	0.347366
**151**	9.583E-05	0.106473
**152**	0.0034858	3.884615
**153**	0.0025025	2.802025
**154**	0.0002208	0.248022
**155**	0.0005192	0.592675
**156**	0.0010517	1.203049
**157**	0.012175	13.97128
**158**	0.0002092	0.239767
**159**	4.083E-05	0.047405
**160**	0.0647975	74.62856
**161**	0.0172833	19.96253
**162**	0.0152792	17.79984
**163**	0.0021767	2.567511
**164**	0.0565442	66.9717
**165**	0.0716633	85.28981
**166**	0.0005558	0.685991
**167**	0.2114575	262.5962
**168**	0.011295	14.10015
**169**	0.04166	52.37515
**170**	0.0205758	26.16477
**171**	5.083E-05	0.066628
**172**	0.0515417	67.3238
**173**	0.1299892	170.9473
**174**	0.001015	1.338352
**175**	0.10917	147.7002
**176**	0.0551767	75.76055
**177**	0.1148283	158.4065
**178**	0.0005192	0.719403
**179**	0.0041317	5.738938
**180**	0.0040567	5.693809
**181**	0.0003758	0.5394
**182**	2.083E-05	0.03012
**183**	0.0002908	0.42536
**184**	2.583E-05	0.037867
**185**	0.0178667	26.26927
**186**	0.1394092	205.8604
**187**	0.0283442	42.01098
**188**	0.0362542	54.10605
**189**	0.0041167	6.243637

**190**	0.0716367	111.7328
**191**	0.0052242	8.236976
**192**	0.0007167	1.145732
**193**	0.0015467	2.502212
**194**	0.0053342	15.92755
**195**	8.333E-07	0.001775
**196**	1.667E-06	0.005965
**197**	0.0000025	0.006598
**198**	2.167E-05	0.064681
**199**	0.0008408	2.545677
**200**	8.333E-07	0.003205
**201**	0.0001367	0.415299
**202**	2.167E-05	0.064923
**203**	1.667E-06	0.005621
**204**	0.0462458	140.6306
**205**	0.0002425	0.737797
**206**	0.3807858	1160.127
**207**	0.0065083	19.83433
**208**	0.0209417	63.91599
**209**	0.00001	0.030707

Table S10. Huang-Rhys (HR) factor and reorganization energies for $[Eu(NO_3)_3(phen)_2]$ per normal vibrational mode.

Normal mode index	Huang-Rhys factor	Reorganization energy / cm^{-1}
**1**	0.002	0.016107728
**2**	0.001	0.012886183
**3**	0.0306667	0.753841693
**4**	0.068	1.862053413
**5**	0.0226667	0.885925067
**6**	0.0076667	0.318933024
**7**	0.0003333	0.012886183
**8**	0.01	0.602429045
**9**	0.0033333	0.206178925
**10**	0.014	1.043780806
**11**	0.0303333	2.309848265
**12**	0.0016667	0.167520376
**13**	0.1683333	17.09352147
**14**	0.0173333	1.868496505
**15**	0.0246667	2.828517123
**16**	0.003	0.373699301
**17**	0	0
**18**	0.0003333	0.032215457
**19**	0.021	2.986372862
**20**	0.0126667	1.810508682
**21**	0.0003333	0.04510164
**22**	0.0146667	2.203537257
**23**	0.202	32.69224574
**24**	0.0443333	7.242034728
**25**	0.0066667	1.159756451
**26**	0.264	48.91594987
**27**	0	0.012886183
**28**	0.0126667	2.374279179
**29**	0.0406667	7.899230051
**30**	0.0196667	4.059147579
**31**	0.0156667	3.244096518
**32**	0.0003333	0.070874005
**33**	0.0023333	0.50900422
**34**	0.006	1.391707741
**35**	0.011	2.809187848
**36**	0.0026667	0.728069328
**37**	0.0283333	7.625398666
**38**	0	0.041880094
**39**	0.0006667	0.202957379
**40**	0.159	64.03144229
**41**	0.0013333	0.525111949
**42**	0.0003333	0.190071196
**43**	0.008	3.311748977

**44**	0.0023333	0.969685255
**45**	0	0.04510164
**46**	0.0003333	0.206178925
**47**	0.0026667	1.29506137
**48**	0.002	1.053445443
**49**	0.0016667	0.879481975
**50**	0	0.009664637
**51**	0.0066667	3.385844528
**52**	0.0806667	42.76601914
**53**	0.0016667	0.972906801
**54**	0.012	6.555845495
**55**	0.0033333	1.949035147
**56**	0.006	3.630682001
**57**	0.0043333	2.744756934
**58**	0.0006667	0.409136304
**59**	0.003	1.96192133
**60**	0.001	0.760284785
**61**	0.0353333	24.93798525
**62**	0.0183333	13.09558326
**63**	0	0.006443091
**64**	0	0.038658548
**65**	0.0003333	0.154634193
**66**	0.0053333	3.746657646
**67**	0.0033333	2.47092555
**68**	0.0003333	0.173963468
**69**	0.0186667	13.69156921
**70**	0.0413333	30.43394221
**71**	0.0026667	2.065010792
**72**	0.0013333	0.947134435
**73**	0.0013333	1.098547083
**74**	0.0093333	7.332238008
**75**	0.1236667	98.81769273
**76**	0.003	2.412937728
**77**	0.0016667	1.43036629
**78**	0	0.086981734
**79**	0.0076667	6.533294675
**80**	0.0003333	0.32215457
**81**	0.0026667	2.187429529
**82**	0.002	1.704197674
**83**	0.0056667	4.806546181
**84**	0.001	0.943912889
**85**	0.007	6.256241745
**86**	0	0.006443091
**87**	0.012	11.21420057
**88**	0.0083333	7.986211784
**89**	0.0043333	3.975387391
**90**	0.1116667	107.3998905
**91**	0.0143333	13.94929287

**92**	0.0173333	17.03875519
**93**	0.0003333	0.441351761
**94**	0.02	19.79317677
**95**	0.0193333	19.52256693
**96**	0.0176667	17.87957862
**97**	0.0243333	25.07651171
**98**	0.025	26.17183725
**99**	0	0.099867917
**100**	0.0183333	19.37759737
**101**	0.0006667	0.628201411
**102**	0.008	8.672401018
**103**	0	0.148191102
**104**	0.0273333	29.2774073
**105**	0	0.161077285
**106**	0.018	19.44847138
**107**	0.092	100.9890145
**108**	0.0003333	0.373699301
**109**	0.0006667	0.927805161
**110**	0.05	57.85896073
**111**	0.014	16.78747463
**112**	0.004	4.864534003
**113**	0.0043333	5.041719017
**114**	0.0093333	11.18520666
**115**	0.0006667	0.895589704
**116**	0.0276667	33.75213427
**117**	0.1943333	243.5198608
**118**	0.0006667	0.856931156
**119**	0.003	3.988273574
**120**	0.0083333	10.87271673
**121**	0.0036667	4.899971006
**122**	0.005	6.700815051
**123**	0.003	4.039818305
**124**	0.0013333	1.920041236
**125**	0.1326667	177.300989
**126**	0.0206667	27.74717309
**127**	0.01	13.65291067
**128**	0.0403333	56.02267968
**129**	0	0
**130**	0.0163333	23.00827937
**131**	0.0016667	2.197094166
**132**	0.0003333	0.393028575
**133**	0.1466667	207.6189556
**134**	0	0.064430914
**135**	0.0406667	58.78354434
**136**	0	0.02255082
**137**	0.0076667	11.35272704
**138**	0.01	15.01884604
**139**	0.0023333	3.611352727

**140**	0.0986667	149.6246899
**141**	0.065	99.01742856
**142**	0.001	1.755742405
**143**	0.0153333	23.50117586
**144**	0.3383333	527.8985857
**145**	0.0006667	1.027673078
**146**	0.0423333	66.51203247
**147**	0.0056667	8.88824458
**148**	0.01	16.23014722
**149**	0.005	7.950774782
**150**	0	0.006443091
**151**	0.108	333.4396443
**152**	0.001	3.434167714
**153**	0	0.041880094
**154**	0	0.012886183
**155**	0.007	22.08369576
**156**	0	0.099867917
**157**	0.0006667	1.810508682
**158**	0.0013333	3.939950388
**159**	0.1863333	578.6508167
**160**	0.0236667	73.17418898
**161**	0.0003333	1.159756451
**162**	0	0.003221546
**163**	0.0496667	154.176734
**164**	0.002	6.6267195
**165**	0.0293333	91.21806643

Table S11. Huang-Rhys (HR) factor and reorganization energies for [Eu(PyrCF$_3$)$_3$(phen)] per normal vibrational mode.

Normal mode index	Huang-Rhys factor	Reorganization
**1**	0.00074	0.00631504
**2**	0.00036	0.00370192
**3**	0	0
**4**	0.00815	0.17333696
**5**	0.00198	0.04975816
**6**	0.02286	0.70434472
**7**	0.00083	0.02645784
**8**	0.00001	0.00032664
**9**	0.00748	0.2841768
**10**	0.00589	0.23616072
**11**	0.00152	0.06391256
**12**	0.00779	0.36028392
**13**	0.00015	0.00729496
**14**	0.00045	0.02580456
**15**	0.00438	0.25869888
**16**	0.00035	0.02134048
**17**	0.00001	0.00043552
**18**	0.00056	0.03691032
**19**	0.00044	0.0293976
**20**	0.00148	0.10757344
**21**	0.00362	0.26327184
**22**	0.00163	0.12455872
**23**	0.00162	0.13130928
**24**	0.00007	0.00631504
**25**	0.00068	0.06064616
**26**	0.00014	0.01371888
**27**	0.0013	0.13130928
**28**	0.04415	4.6263112
**29**	0.00046	0.0484516
**30**	0	0.00032664
**31**	0.00108	0.13740656
**32**	0.00007	0.0097992
**33**	0.00027	0.03952344
**34**	0.00074	0.11563056
**35**	0.00002	0.002722
**36**	0.01203	1.99511712
**37**	0.0012	0.20828744
**38**	0.00061	0.11073096
**39**	0.00211	0.39730312
**40**	0.00853	1.66216208
**41**	0.01447	2.95184568
**42**	0.00033	0.06924768
**43**	0.00003	0.0059884
**44**	0.00041	0.0903704
**45**	0.00023	0.0549844

**46**	0.0008	0.1916288
**47**	0.00038	0.09298352
**48**	0.05508	14.45240456
**49**	0.00746	1.9603844
**50**	0.03918	10.52303424
**51**	0.0032	0.88377896
**52**	0.00104	0.29049184
**53**	0.001	0.28450344
**54**	0.00034	0.10049624
**55**	0.00291	0.8721288
**56**	0.00366	1.11373352
**57**	0.00177	0.53939152
**58**	0.02358	7.8970664
**59**	0.00001	0.00348416
**60**	0.00144	0.48745576
**61**	0.00002	0.00783936
**62**	0.00016	0.05563768
**63**	0.00277	1.02641176
**64**	0.01899	7.46448616
**65**	0.02472	9.75934992
**66**	0.00571	2.4057036
**67**	0.00008	0.03516824
**68**	0.0005	0.21590904
**69**	0.00134	0.58435896
**70**	0.00333	1.53999872
**71**	0.00434	2.05325904
**72**	0.01185	5.8071148
**73**	0.0004	0.20240792
**74**	0.00012	0.06173496
**75**	0.00806	4.216378
**76**	0.00087	0.48386272
**77**	0.00001	0.0070772
**78**	0.00081	0.46012688
**79**	0.00022	0.127934
**80**	0.01678	9.69500184
**81**	0.04019	23.319374
**82**	0.00785	4.608346
**83**	0.001	0.6010176
**84**	0.00038	0.23060784
**85**	0.00126	0.76640632
**86**	0.00358	2.25305384
**87**	0.01215	7.6847504
**88**	0.00092	0.58936744
**89**	0.00023	0.14840344
**90**	0.00079	0.5318788
**91**	0.00031	0.21188048
**92**	0.00153	1.03359784
**93**	0.00034	0.228648

**94**	0.00001	0.00740384
**95**	0.00209	1.5101656
**96**	0.00512	3.7351284
**97**	0.02086	15.27891264
**98**	0.14474	106.08995
**99**	0.00952	7.01611832
**100**	0.00206	1.52018256
**101**	0.00476	3.6725224
**102**	0.0018	1.40248328
**103**	0.0003	0.23746728
**104**	0.00677	5.38063184
**105**	0.00001	0.0070772
**106**	0.00093	0.7414728
**107**	0.00086	0.68605288
**108**	0.00666	5.42668808
**109**	0.00044	0.35636424
**110**	0.00545	4.4744236
**111**	0.00091	0.76422872
**112**	0.00018	0.15417408
**113**	0.00043	0.35897736
**114**	0.1308	112.8311463
**115**	0.00541	4.68064232
**116**	0.00273	2.36814
**117**	0.00082	0.73047592
**118**	0.00019	0.16517096
**119**	0.00001	0.00881928
**120**	0.0024	2.16899848
**121**	0.00247	2.25076736
**122**	0.00016	0.14252392
**123**	0.00308	2.83022672
**124**	0.00001	0.01371888
**125**	0.00039	0.37411168
**126**	0.00646	6.22227424
**127**	0.00275	2.68095224
**128**	0.00091	0.91600744
**129**	0.00141	1.42643688
**130**	0.12306	125.2915913
**131**	0.00097	0.99450992
**132**	0.04641	48.10895464
**133**	0.00356	3.70823504
**134**	0.00218	2.27047464
**135**	0.15553	167.7462986
**136**	0.0518	56.08289032
**137**	0.00934	10.12279136
**138**	0.001	1.09413512
**139**	0.00004	0.03876128
**140**	0.0003	0.32653112
**141**	0.00002	0.02591344

**142**	0.0016	1.77997024
**143**	0.00015	0.1616868
**144**	0.00067	0.75410288
**145**	0.00002	0.01861848
**146**	0.00012	0.14121736
**147**	0.11578	135.5849976
**148**	0.03927	46.04785624
**149**	0.36724	433.3871497
**150**	0.02976	35.3838224
**151**	0.00032	0.38663288
**152**	0.02006	24.21534752
**153**	0.0041	4.95697976
**154**	0.00014	0.16974392
**155**	0.04405	54.1149932
**156**	0.00001	0.01644088
**157**	0	0
**158**	0	0.00032664
**159**	0.02029	25.22564504
**160**	0.00268	3.36428312
**161**	0.00091	1.15184152
**162**	0.0008	1.0212944
**163**	0.00101	1.29316776
**164**	0.05942	77.6875132
**165**	0.24058	314.9973527
**166**	0.00108	1.42229944
**167**	0.00187	2.4574216
**168**	0.01693	22.29024024
**169**	0.00254	3.39302744
**170**	0.0047	6.29794584
**171**	0.00569	7.63597216
**172**	0.00701	9.41681344
**173**	0.13747	185.1252887
**174**	0.01369	18.52233896
**175**	0.01098	14.8681084
**176**	0.00537	7.32305104
**177**	0.01956	26.844364
**178**	0.00074	1.03610208
**179**	0.00801	11.40659544
**180**	0.00736	10.54470136
**181**	0.00086	1.23644128
**182**	0.00566	8.15271664
**183**	0.00029	0.41842584
**184**	0	0.00555288
**185**	0.08935	130.2529262
**186**	0.00514	7.78644432
**187**	0.25549	395.082839
**188**	0.00488	7.57184184
**189**	0.00118	1.829184

**190**	0.01877	29.17679136
**191**	0.00038	0.59862224
**192**	0.00079	1.22664208
**193**	0.00038	0.58773424
**194**	0.02755	43.36984376
**195**	0.00021	0.33143072
**196**	0.00006	0.08743064
**197**	0.00076	1.19299816
**198**	0.03678	58.27453584
**199**	0.03037	48.23122688
**200**	0.00002	0.03745472
**201**	0.00449	7.14546776
**202**	0.00001	0.02090496
**203**	0.00319	5.11779552
**204**	0.0843	135.3355535
**205**	0.07889	127.6819428
**206**	0.12977	210.9688278
**207**	0.01603	26.123034
**208**	0.00043	0.70543352
**209**	0.00079	1.30579784
**210**	0.00107	1.77539728
**211**	0.0021	3.51823944
**212**	0.0292	49.11326376
**213**	0.00748	12.58892336
**214**	0.05853	100.1132002
**215**	0.02316	39.86782744
**216**	0.01292	22.29840624
**217**	0	0.00010888
**218**	0.15252	264.7424822
**219**	0.00002	0.03037752
**220**	0.00123	2.15876376
**221**	0.00001	0.01600536
**222**	0.00597	10.7508112
**223**	0.00146	2.64774384
**224**	0.1223	223.7009283
**225**	0.0002	0.37030088
**226**	0.00116	2.18979456
**227**	0	0.00032664
**228**	0	0.00010888
**229**	0.00002	0.07044536
**230**	0	0.00021776
**231**	0.00002	0.05182688
**232**	0.00003	0.09483448
**233**	0	0
**234**	0	0.0092548
**235**	0.00001	0.03614816
**236**	0.00001	0.02406248
**237**	0.00005	0.16125128

**238**	0.00578	20.0056112
**239**	0.00152	5.2817688
**240**	0.0598	207.7825634
**241**	0.0017	5.93461328
**242**	0.01347	47.03964416
**243**	0.00726	25.46659648
**244**	0.00007	0.23659624
**245**	0.00001	0.0370192
**246**	0.00063	2.24423456
**247**	0.00029	1.03098472
**248**	0.00003	0.08906384
**249**	0	0
**250**	0.03853	136.8987437
**251**	0.00055	1.96060216
**252**	0.00141	5.02905832

The vibrational overlap integrals for harmonic oscillators with a sufficient low frequency change have the form expressed in Eq. S34. In this equation a massive dependency of the Huang-Rhys factor (S_j) is found, where it can be defined by Eq. S35, in which M_j is the reduced mass, ω_j the vibrational energy, $\hbar$ Planck's reduced constant and $\Delta Q_{e,j}$ the normal mode displacement.[37]

$$\langle \bar{\chi}_j | \chi_j \rangle = exp(-S_j) S_j^{(\bar{\nu}_j - \nu_j)} \frac{\nu_j!}{\bar{\nu}_j!} \left[\sum_{0}^{\nu_j} \frac{\bar{\nu}_j! \, (-S_j)^{\nu}}{(\nu_j - \nu)! \, (\bar{\nu}_j - \nu_j + \nu)! \, \nu!} \right]^2 \quad \text{(S34)}$$

$$S_j = \frac{1}{2} \left(\frac{M_j \omega_j}{\hbar} \right) (\Delta Q_{e,j})^2 \quad \text{(S35)}$$

The other property we used to analyze the vibronic coupling is the reorganization energy per vibrational modes, which can be correlated to the Huang-Rhys factor (Eq. S36):

$$\lambda_j = S_j \hbar \omega_j \quad \text{(S36)}$$

It should be emphasized that in this formulation, the reorganization energy explicitly accounts for final-state dynamics, thereby incorporating vibronic details by considering the difference between points of each potential energy surfaces.

The relationship between the vibrational modes of S_1 and T_1 electronic states is depicted in Figure S11 by the Duschinsky rotation matrix. It should be noted that for the calculation of the intersystem crossing rates from the correlation function approach we used the reorganization energies per normal mode, determined from Eq. S36. These energies were directly determined in *Orca_ESD* software from the Hessian matrices of the S_1 and T_1 states. The shape of the correlation function dictates the behavior of vibrational dynamics; therefore, the analysis is reported in the main text and Figure S12.

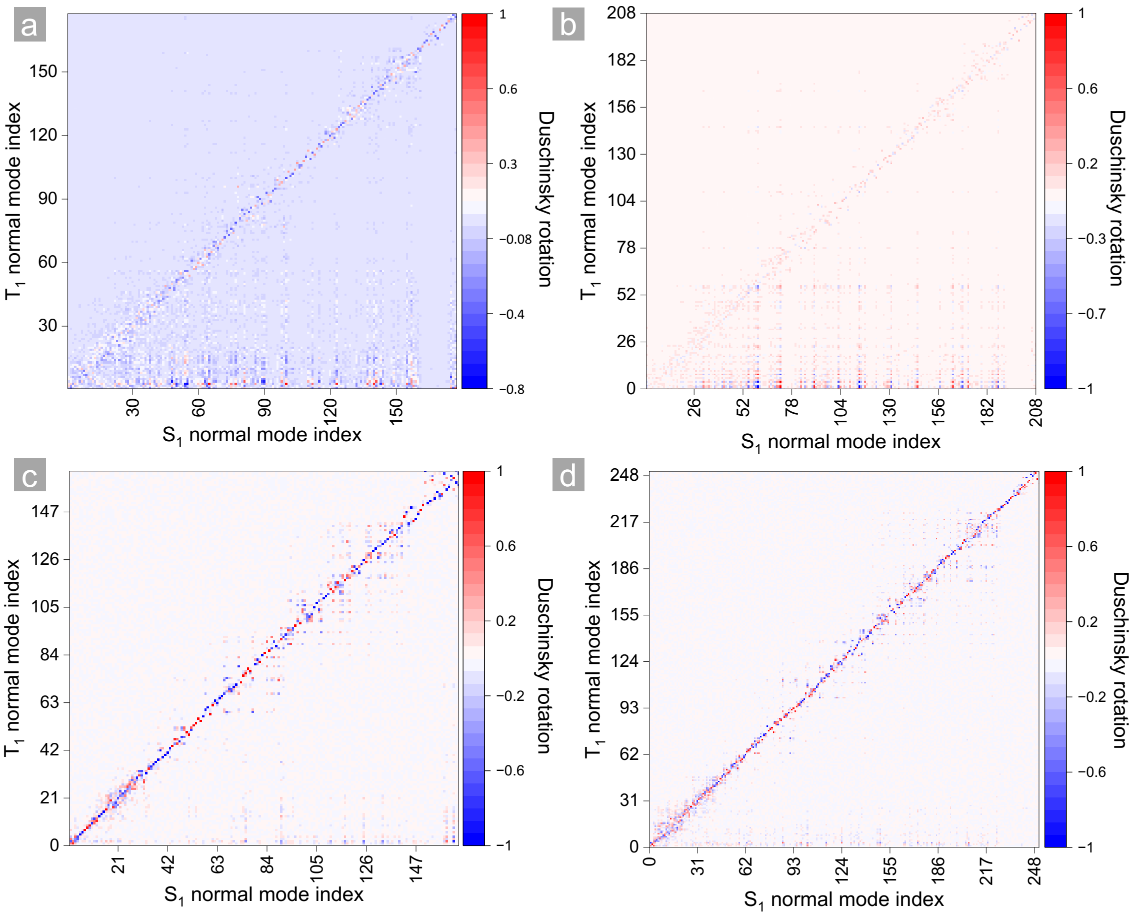

Figure S11. Duschinsky rotation matrix between the S_1 and T_1 normal modes of (a) $[Eu(tta)_3(H_2O)_2]$, (b) $[Eu(tta)_4]^-$, (c) $[Eu(NO_3)_3(phen)_2]$ and (d) $[Eu(PyrCF_3)_3(phen)]$ complexes.

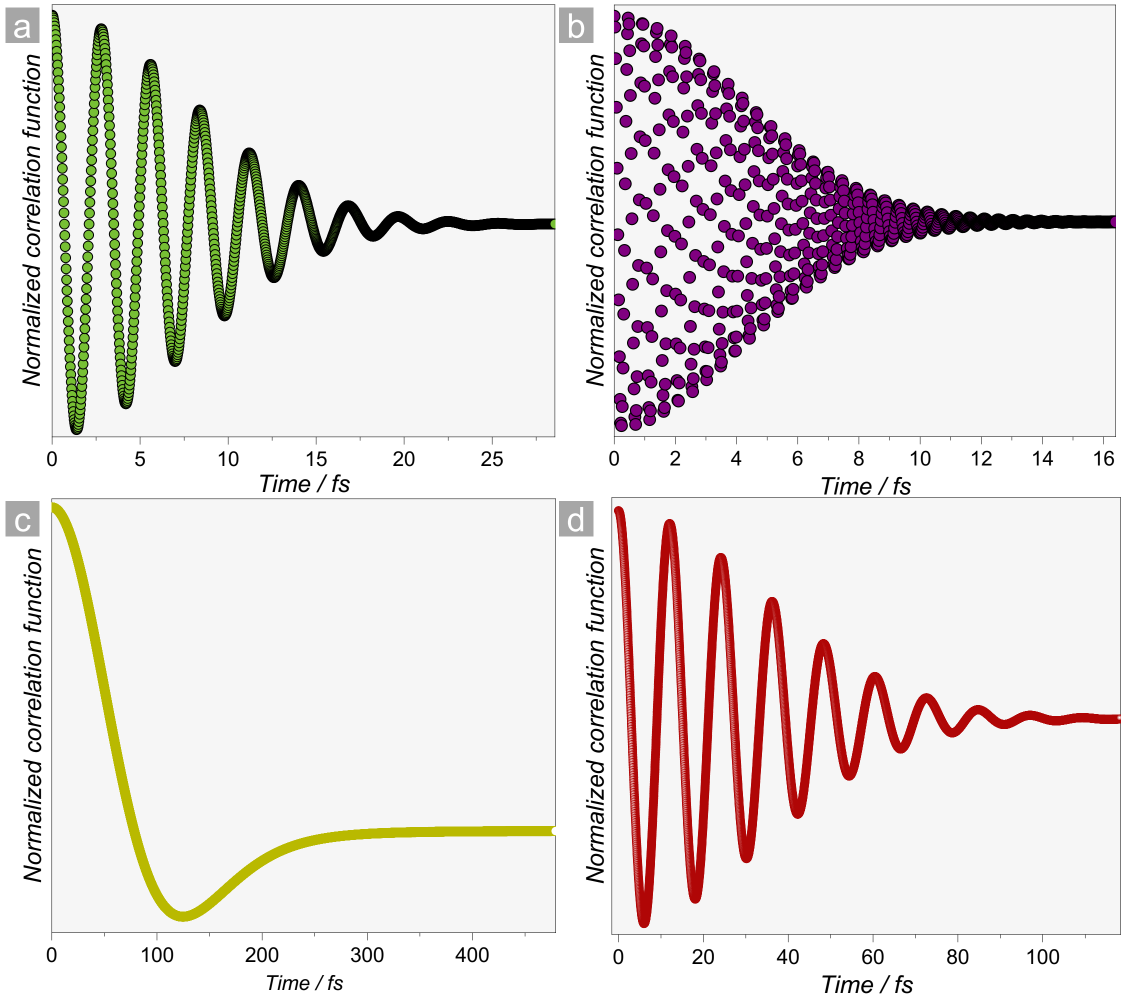

Figure S12. Correlation function for the vibrational density of states of (a) [Eu(tta)$_3$(H$_2$O)$_2$], (b) [Eu(tta)$_4$]$^-$, (c) [Eu(NO$_3$)$_3$(phen)$_2$], and (d) [Eu(PyrCF$_3$)$_3$(phen)] complexes.

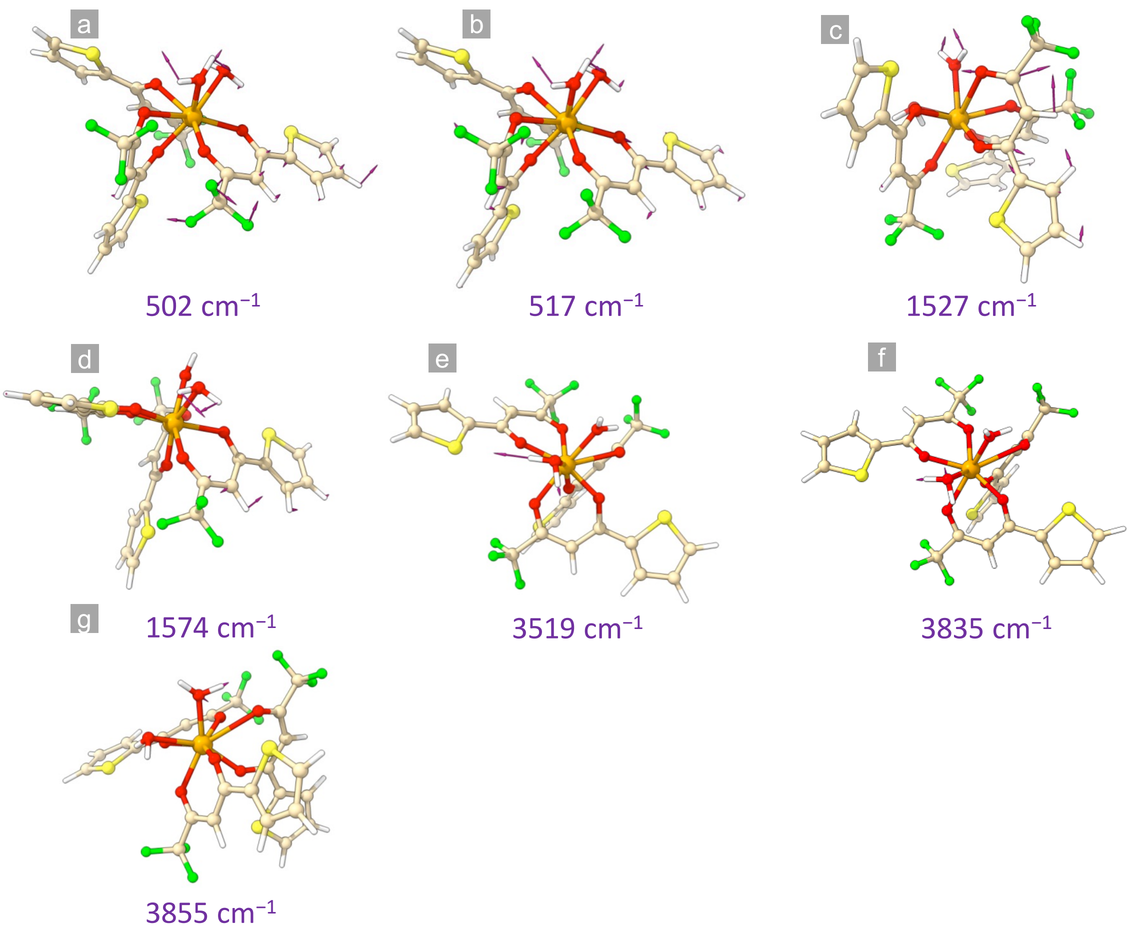

Figure S13. Normal vibrational modes that were decomposed into LVM for the $[Eu(tta)_3(H_2O)_2]$ complex. The vibrational frequencies are as follows: (a) 502 cm^{-1}, (b) 517 cm^{-1}, (c) 1527 cm^{-1}, (d) 1574 cm^{-1}, (e) 3519 cm^{-1}, (f) 3835 cm^{-1}, (g) 3855 cm^{-1}.

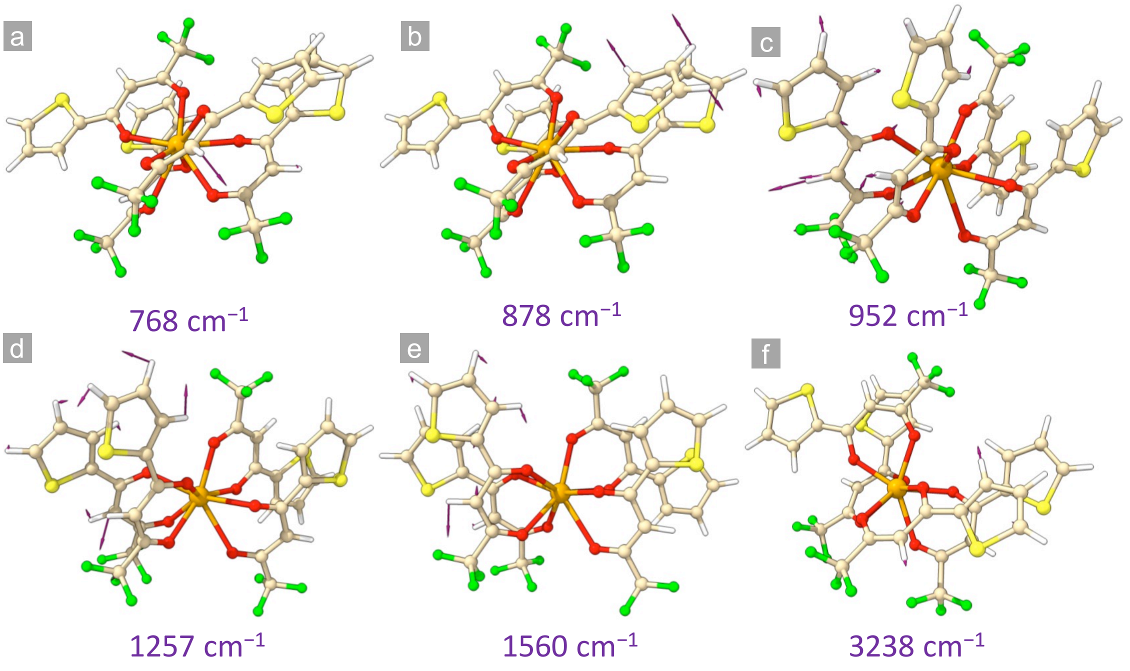

Figure S14. Normal vibrational modes of $[Eu(tta)_4]^-$ complex that were decomposed into LVM. The vibrational frequencies are as follows: (a) 768 cm^{-1}, (b) 878 cm^{-1}, (c) 952 cm^{-1}, (d) 1257 cm^{-1}, (e) 1560 cm^{-1}, (f) 3283 cm^{-1}.

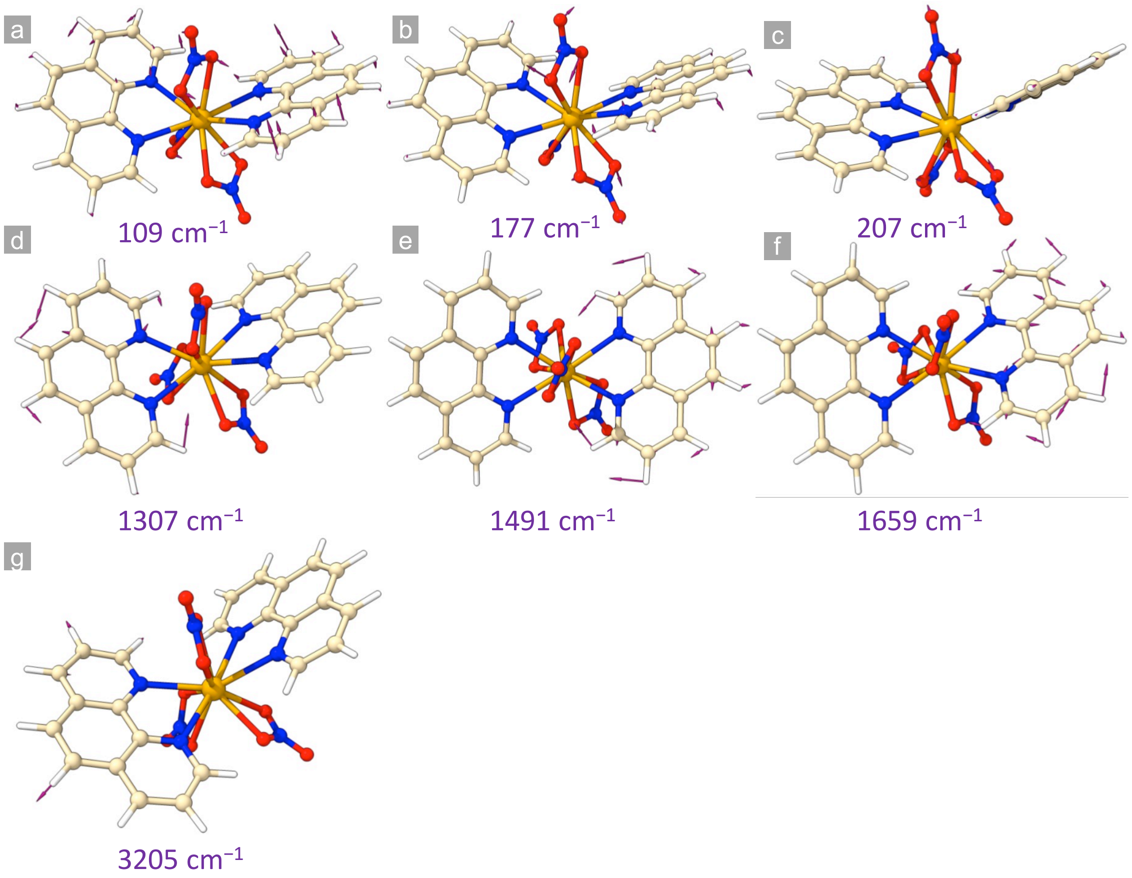

Figure S15. Normal vibrational modes of $[Eu(NO_3)_3(phen)_2]$ complex that were decomposed into LVM. The vibrational frequencies are as follows: (a) 109 cm^{-1}, (b) 177 cm^{-1}, (c) 207 cm^{-1}, (d) 1307 cm^{-1}, (e) 1491 cm^{-1}, (f) 1659 cm^{-1}, (g) 3205 cm^{-1}.

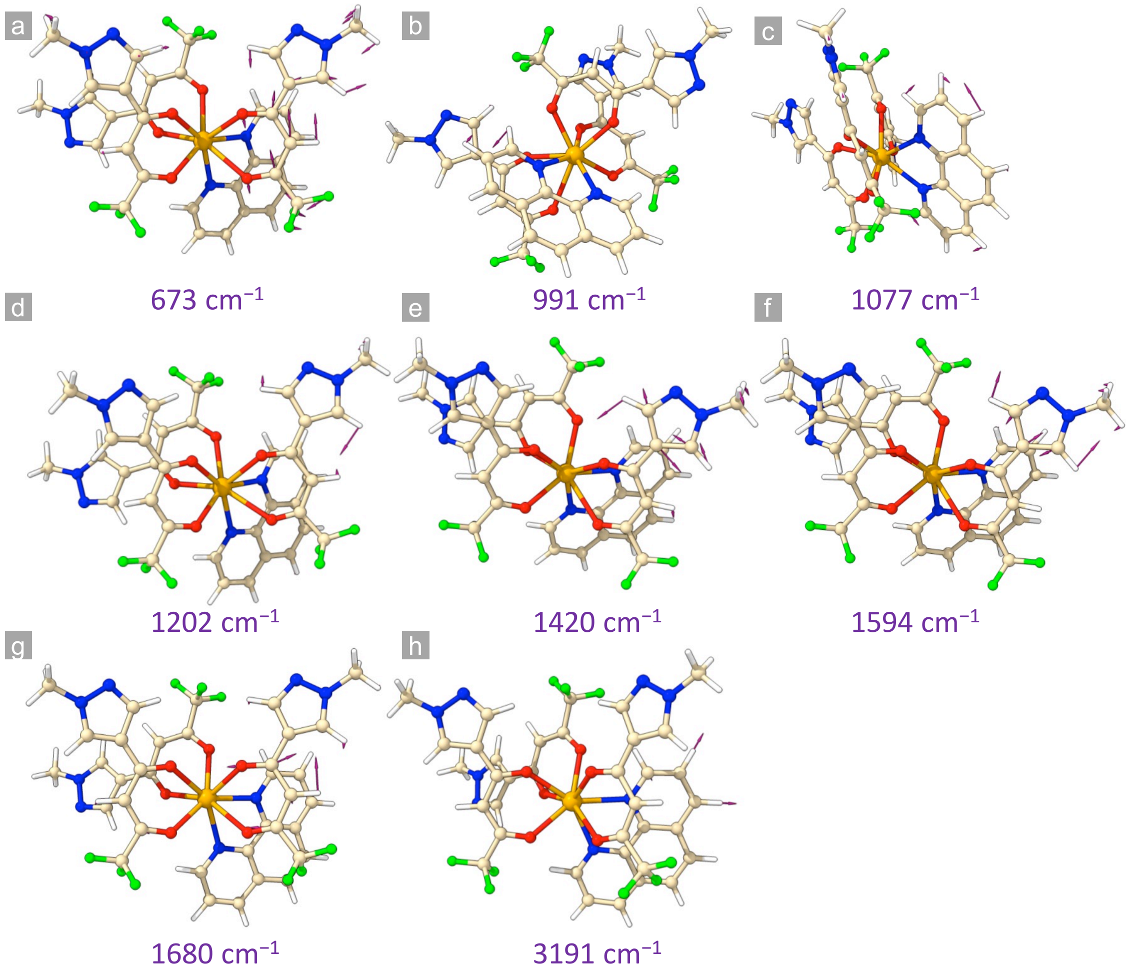

Figure S16. Normal vibrational modes of [Eu(PyrCF$_3$)$_3$(phen)] complex that were decomposed into LVM. The vibrational frequencies are as follows: (a) 673 cm^{-1}, (b) 991 cm^{-1}, (c) 1077 cm^{-1}, (d) 1202 cm^{-1}, (e) 1420 cm^{-1}, (f) 1594 cm^{-1}, (g) 1680 cm^{-1}, (h) 3191 cm^{-1}.

References

[1] F. Neese, Software update: The Orca program system – Version 5.0, *Wires Comp. Mol. Sci*. **2022**, 12, 5, e1606.

[2] A. D. Becke, Density-functional thermochemistry III. The role of exact exchange, *J. Chem. Phys*. **1992**, 98, 5648 – 5652.

[3] E. Caldewyher, J-M. Lewis, S. Ehlert, S. Grimme, Extension and evaluation of the D4 London-dispersion model for periodic systems, *Phys. Chem. Chem. Phys*. **2020**, 22, 8499 – 8512.

[4] F. Weigend, R. Ahlrichs, Balanced basis sets of split valence, triple zeta valence and quadruple zeta valence quality for H to Rn: Design and assessment of accuracy, *Phys. Chem. Chem. Phys*. **2005**, 7, 3297 – 3305.

[5] M. Dolg, H. Stoll, A. Savin, H. Preuss, Energy-adjusted pseudopotentials for the rare earth elements, *Theoret. Chim. Acta* **1989**, 75, 173 – 194.

[6] M. Dolg, H. Stoll, H. Preuss, A combination of quasirelativistic pseudopotential and ligand field calculations for lanthanoid compounds, *Theoret. Chim. Acta* **1993**, 85, 441 – 450.

[7] T. Froizheim, S. Grimme, J-M. Mewes, Either Accurate Singlet-Triplet Gaps for Excited-State Structures: Testing and Understanding the Performance of TD-DFT for TADF emitters, *J. Chem. Theory Comp*. **2022**, 18, 7702 – 7713.

[8] J-D. Chai, M. Head-Gordon, Long-range corrected hybrid density functionals with damped atom-atom dispersion corrections, *Phys. Chem. Chem. Phys*. **2008**, 10, 6615 – 6620

[9] P. Verma, J. Autschbach, *J. Chem. Theory and Comp*. **2012**, 9, 1052 – 1067.

[10] T. Nakajima, K. Hirao, The Douglas-Kroll-Hess Approach, *Chem. Rev*. **2012**, 112, 385 – 402.

[11] D. Aravena, F. Neese, D. A. Pantazis, Improved Segmented All-Electron Relativistically Contracted Basis Sets for the Lanthanides, *J. Chem. Theory Comput*. **2016**, 12, 1148 – 1156.

[12] F. Neese, Efficient and accurate approximations to the molecular spin-orbit coupling operator and their use in molecular g-tensor calculations, *J. Chem. Phys*. **2005**, 122, 034107.

[13] B. de Souza, F. Neese, R. Izsák, On the theoretical prediction of fluorescence rates from first principles using the path integral approach, *J. Chem. Phys*. **2018**, 148, 034104.

B. de Souza, G. Farias, F. Neese, R. Izsák, Predicting Phosphorescence Rates of Light Organic Molecules Using Time-Dependent Density Functional Theory and the Path Integral Approach to Dynamics, *J. Chem. Theory Comp*. **2019**, 15, 1896 – 1904.

[15] P. K. Samanta, D. Kim, V. Coropceanu, J-L. Brédas, Up-conversion Intersystem Crossing Rates in Organic Emitters for Thermally Activated Delayed Fluorescence: Impact of the Nature of Singlet vs Triplet Excited States, *J. Am. Chem. Soc.* **2017**, 139, 11, 4042 – 4051.

[16] S. V. Rao, M. Piccardo, A. Soncini, Study of the most relevant spin-orbit coupling descriptions of magnetic excitations in a series of lanthanide complexes, *Phys. Chem. Chem. Phys*. **2022**, 24, 9007 – 9017.

[17] M. Gualland, F. Riobé, J. Ouyang, N. Saleh, F. Pointillart, V. Dorcet, B. Le Guennic, O. Cador, J. Crassous, C. Andraud, C. Monnerau, O. Maury, Helicenic Complexes of Lanthanides: Influence of the f-Element on the Intersystem Crossing Efficiency and Competition between Luminescence and Oxygen Sensitization, *Euro. J. Inorg. Chem*. **2019**, 2019, 118 – 125.

[18] V. Rai-Constapel, M. Etinski, C. M. Marian, Photophysics of Xanthone: A Quantum Chemical Perusal, *J. Phys. Chem. A* **2013**, 117, 19, 3935 – 3944.

[19] R. Ahmed, A. K. Manna, Origins of Molecular-Twist-Triggered Intersystem Crossing in Functional Perylenediimides: Singlet-Triplet Gap versus Spin-Orbit Coupling, *J. Phys. Chem. A* **2022**, 126, 38, 6594 – 6603.

[20] A. N. Carneiro Neto, R. T. Moura Jr., L. D. Carlos, O. L. Malta, M. Sanadar, A. Melchior, E. Kraka, S. Ruggieri, M. Bettinelli, F. Piccinelli, Dynamics of the Energy Transfer Process in Eu(III) Complexes Containing Polydentate Ligands Based on Pyridine, Quinoline, and Isoquinoline as Chromophoric Antennae, *Inorg. Chem.* **2022**, 61, 41, 16333 – 16346.

[21] H. La Force, E. Kraka, Characterizing guanine's binding modes with potential Ru(II) monofunctional adducts: A local vibrational mode study, *Chem. Phys. Lett*. **2023**, 828, 140733.

[22] E. Kraka, M. Quintano, H. W. La Force, J. J. Antonio, M. Freindorf, The Local Vibrational Mode Theory and Its Place in the Vibrational Spectroscopy, *J. Phys. Chem. A* **2022**, 126, 8781 – 8798.

[23] R. T. Moura Jr, M. Quintano, C. V. Santos Jr, V. A. C. A. Albuquerque, E. C. Aguiar, E. Kraka, A. N. Carneiro Neto, Featuring a new computational protocol for the estimation of intensity and overall quantum yield in lanthanide chelates with applications to Eu(III) mercapto-triazole Schiff base ligands, *Opt. Mat. X* **2022**, 16, 100216.

[24] R. T. Moura Jr, M. Quintano, J. J. Antonio, M. Freindorf, E. Kraka, Automatic Generation of Local Vibrational Mode Parameters: From Small to Large Molecules and QM/MM Systems, *J. Phys. Chem. A* **2022**, 126, 9313 – 9331.

[25] W. Zou, Y. Tao, M. Freindorf, M. Z. Makos, N. Verma, D. Cremer, E. Kraka, Local Vibrational Mode Analysis (lmodea), Computational and Theoretical Chemistry Group (CATCO), Southern Methodist University, Dallas, TX, USA (2022).

[26] T. J. Penfold, E. Gindensperger, C. Daniel, C. M. Marian, Spin-Vibronic Mechanism for Intersystem Crossing, *Chem. Rev*. **2018**, 118, 6975 – 7025.

[27] R. Ianconescu, E. Polak, Photoinduced Cooling of Polyatomic Molecules in Electronically Excited State in the Presence of Duschinskii Roations, *J. Phys. Chem. A* **2004**, 108, 7778 – 7784.

[28] Q. Peng, Y. Niu, Q. Shi, X. Gao, Z. Shuai, Correlation Function Formalism for Triplet Excited State Decays: Combined Spin-Orbit and Nonadiabatic Couplings, *J. Chem. Theory Comput*. **2013**, 9, 2, 1132 – 1143.

[29] Q. Peng, Y. Niu, C. Deng, Z. Shuai, Vibration correlation function formalism of radiative and non-radiative rates for complex molecules, *Chem. Phys*. **2010**, 370, 215 – 222.

[30] A. Baiardi, J. Bloino, V. Barone, General Time Dependent Approach to Vibronic Spectroscopy Including Franck-Condon, Herzberg-Teller, and Duschinsky Effects, *J. Chem. Theory and Comp*. **2013**, 9, 4097 – 4115.

[31] S. Majumdar, H. S. Majumdar, R. Österbacka, E. McCarthy, *Organic Spintronics*, in: Comprehensive Nanoscience and Technology, 2016.

[32] E. Bright Wilson Jr., Some Mathematical Methods for the Study of Molecular Vibrations, *J. Chem. Phys*. **1941**, 9, 76 – 84.

[33] Z. Konkoli, D. Cremer, A new way of analyzing vibrational spectra. I. Derivation of adiabatic internal modes, *Int. J. Quantum Chem*. **1998**, 67, 1 – 9.

[34] M. Quintano, A. A. A. Delgado, R. T. Moura Jr, M. Freindorf, E. Kraka, Local mode analysis of characteristic vibrational coupling in nucleobases and Watson-Crick base pairs of DNA, *Electron. Struc*. **2022**, 4, 044005.

[35] W. Zou, R. Kalescky, E. Kraka, D. Cremer, Relating normal vibrational modes to local vibrational modes with the help of an adiabatic connection scheme, *J. Chem. Phys*. **2012**, 167, 084114.

[36] R. Marin, G. Brunet, M. Murugesu, Shining New Light on Multifunctional Lanthanide Single-Molecule Magnets, *Angew. Chemie* **2021**, 60, 4, 1728 – 1746.

[37] M. de Jong, L. Seijo, A. Meijerink, F. T. Rabouw, Resolving the ambiguity in the relation between Stokes shift and Huang-Rhys parameters, *Phys. Chem. Chem. Phys.* **2015**, 17, 16959 – 16969.
